# Hepatocyte *Period 1* dictates oxidative substrate selection independent of the core circadian clock

**DOI:** 10.1016/j.celrep.2024.114865

**Published:** 2024-10-16

**Authors:** Jiameng Sun, Yiming Zhang, Joshua A. Adams, Cassandra B. Higgins, Shannon C. Kelly, Hao Zhang, Kevin Y. Cho, Ulysses G. Johnson, Benjamin M. Swarts, Shun-lchi Wada, Gary J. Patti, Leah P. Shriver, Brian N. Finck, Erik D. Herzog, Brian J. DeBosch

**Affiliations:** 1 Department of Pediatrics, Washington University School of Medicine, St. Louis, MO 63110, USA; 2 Department of Chemistry, Washington University in St. Louis, St. Louis, MO 63130, USA; 3 Center for Metabolomics and Isotope Tracing, Washington University in St. Louis, St. Louis, MO 63130, USA; 4 Department of Medicine, Washington University School of Medicine, St. Louis, MO 63110, USA; 5 Department of Chemistry and Biochemistry, Central Michigan University, Mount Pleasant, MI 48859, USA; 6 Biochemistry, Cellular, and Molecular Biology Program, Central Michigan University, Mount Pleasant, MI, USA; 7 Institute of Microbial Chemistry (BIKAKEN), 3-14-23 Kamiosaki, Shinagawa-ku, Tokyo 141-0021, Japan; 8 Department of Biology, Washington University in St. Louis, St. Louis, MO 63130, USA; 9 Department of Cell Biology & Physiology, Washington University School of Medicine, St. Louis, MO 63110, USA; 10 Lead contact

## Abstract

Organisms integrate circadian and metabolic signals to optimize substrate selection to survive starvation, yet precisely how this occurs is unclear. Here, we show that hepatocyte *Period 1* (*Per1*) is selectively induced during fasting, and mice lacking hepatocyte *Per1* fail to initiate autophagic flux, ketogenesis, and lipid accumulation. Transcriptomic analyses show failed induction of the fasting hepatokine *Fgf21* in *Per1*-deficient mice, and single-nucleus multiome sequencing defines a putative responding hepatocyte subpopulation that fails to induce the chromatin accessibility near the *Fgf21* locus. *In vivo* isotopic tracing and indirect calorimetry demonstrate that hepatocyte *Per1*-deficient mice fail to transit from oxidation of glucose to fat, which is completely reversible by exogenous FGF21 or by inhibiting pyruvate dehydrogenase. Strikingly, disturbing other core circadian genes does not perturb *Per1* induction during fasting. We thus describe *Per1* as an important mechanism by which hepatocytes integrate internal circadian rhythm and external nutrition signals to facilitate proper fuel utilization.

## In brief

In this paper, Sun et al. show that a core circadian gene, *Period 1,* is uniquely upregulated during fasting in liver. Mice lacking hepatocyte *Per1* fail to oxidize glucose and fatty acid properly during fasting, a defect rescued by supplying FGF21 or inhibiting pyruvate dehydrogenase activity.

## Graphical abstract

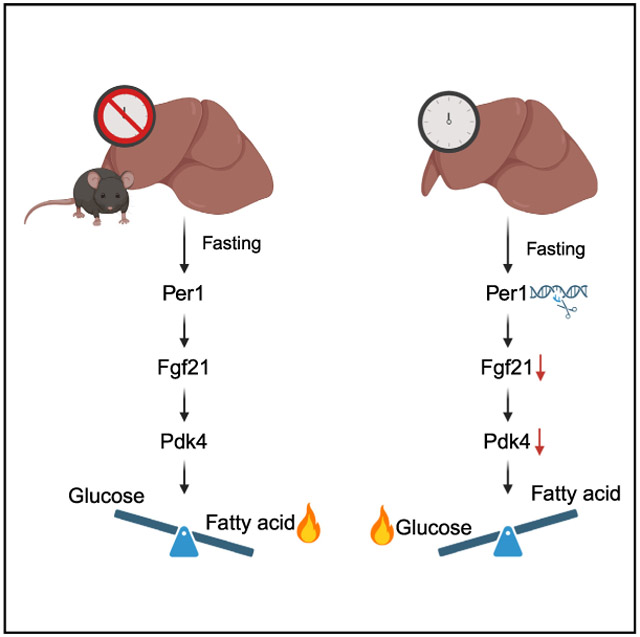

## INTRODUCTION

Proper fuel selection during fasting or famine is a matter of survival. Hepatocytes reside at the intersection of portal and venous circulations and thus moderate acute and long-term energy homeostasis by coordinating substrate selection within the liver and across organ systems.^1^ Whereas the post-prandial state is marked by meal-derived carbohydrate flux from the portal system into hepatocytes and subsequent glucose oxidation through the tricarboxylic acid (TCA) cycle,^2^ the hallmarks of fasting consist of adaptations that include a transition from exogenous glucose and glycogen-derived glucose oxidation to fatty acid β-oxidation. This elegantly coordinated process comprises peripheral lipolysis and lipid export to fuel hepatocyte oxidation, ketogenesis, and storage of excess lipid as intrahepatic triglyceride for rapid on-site mobilization.^3-7^ Fasting hepatocytes further compensate by inducing autophagic flux to salvage organelles and glycoproteins as substrate to fuel gluconeogenesis and ketogenesis.^8,9^ Finally, fasting hepatocytes communicate and integrate extrahepatic fat oxidation and promote peripheral insulin sensitization by secreting fibroblast growth factor 21 (FGF21), an anti-diabetic hepatokine that mediates efficient substrate absorption during the next refeeding cycle.^10-14^ Together, compensatory actions in the hepatocyte maintain survival during shorter and longer-term fasting by sustaining carbon flux to key organs and by altering chromatin structure to prepare for prolonged or repeated famine.^15-17^

**Figure 1.**
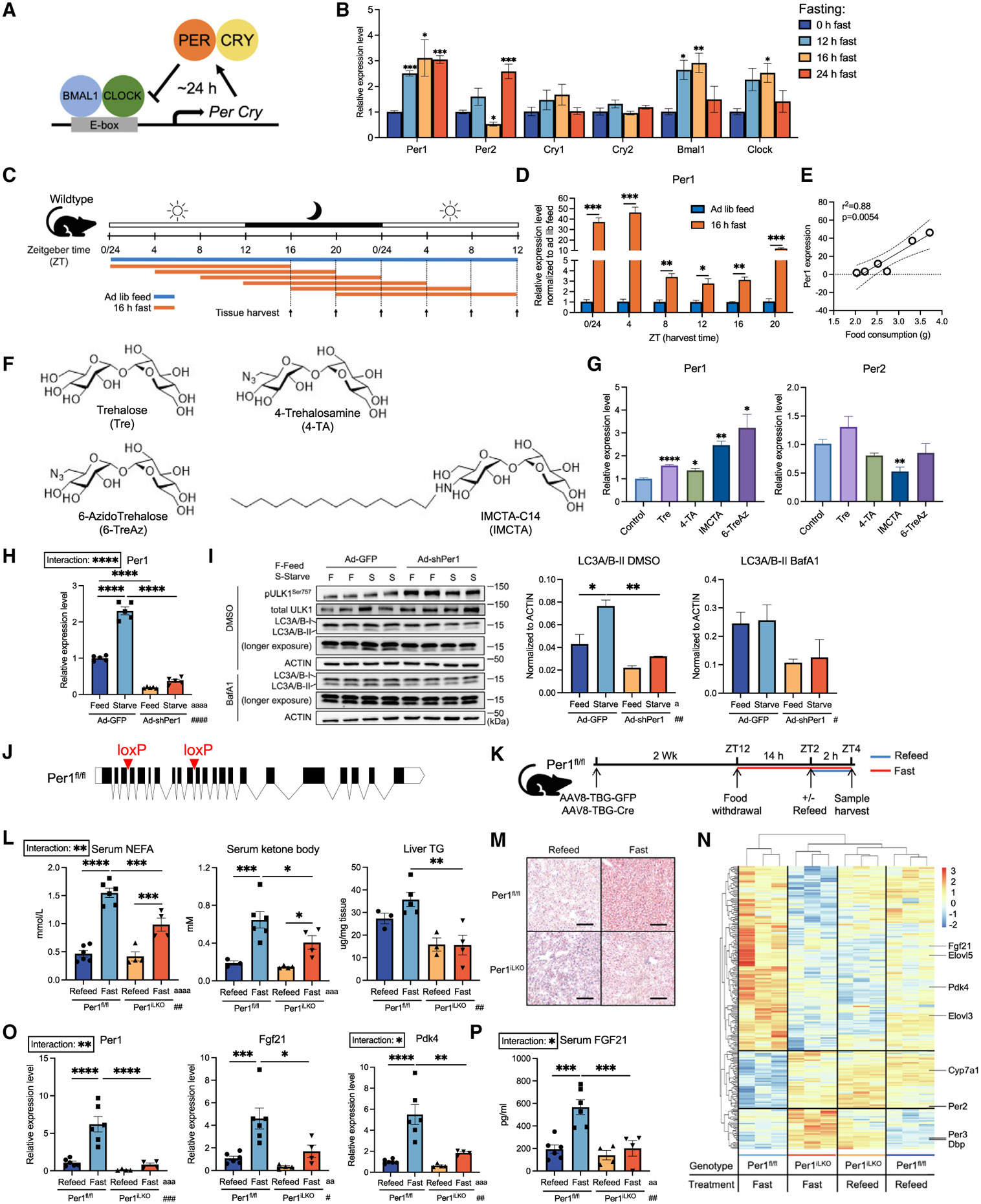
Hepatocyte *Per1* mediates canonical physiological fasting responses (A) Schematic of the canonical core circadian regulatory network. (B) Relative expression level of circadian genes in liver from mice fasted for 0, 12, 16, or 24 h; *n* = 3. (C) Schematic of the 24-h time-course harvest of tissues from mice either fed *ad libitum* or fasted for 16 h. (D) Relative expression level of *Per1* (normalized to mice fed *ad libitum* harvested at the same time) in liver harvested throughout the 24-h time course; *n* = 3–4. (E) Correlation test result between normalized expression level of *Per1* in 16-h-fasted mice in (D) and the corresponding food consumption measured within the same period in the mice fed *ad libitum*. Dotted line denotes the 95% confidence interval for the simple linear regression calculation. (F) Chair conformations of trehalose (Tre), 6-azido trehalose (6-TreAz), 4-trehalosamine (4-TA), and IMCAT-C14 (IMCTA). (G) Relative expression level of *Per1* and *Per2* from isolated wild-type primary hepatocytes treated for 24 h with regular growth medium (Control), Tre (100 mM), 6-TreAz (100 mM), 4-TA (100 mM), or IMCTA (100 μM) in complete culture medium; *n* = 3–6. (H) Relative expression level of *Per1* from AML12 cells transduced with Ad-GFP or Ad-shPer1 for 48 h followed by a full medium change to either complete or starvation medium for 6 h; *n* = 5. (I) Immunoblot analysis of AML12 cells described in (H) with additional treatment of dimethyl sulfoxide (DMSO) or bafilomycin A1 (BafA1) (200 nM) (left) during the medium change, and quantification of LC3A/B-II (right); *n* = 2. (J) *Per1*^fl/fl^ mice design at the *Per1* locus. (K) Schematic of the 14 h + 2 h fast/refeed experimental design in *Per1*^fl/fl^ (*Per1*^fl/fl^, AAV8-TBG-GFP) and *Per1*^iLKO^ (*Per1*^fl/fl^, AAV8-TBG-Cre) mice. (L) Serum non-esterified fatty acid (NEFA) (left), ketone body (middle), and hepatic triglyceride (TG) level from mice in (K); *n* = 4–6. (M) Representative images from oil red O (ORO)-stained liver tissue in (K). Scale bar, 100 μm. (N) Unsupervised hierarchical clustering of all differentially regulated genes (*p* < 0.05) from bulk RNA sequencing in liver harvested from (K); *n* = 3. (O) Relative expression level of *Per1, Fgf21*, and *Pdk4* in liver harvested from mice in (K); *n* = 4–6. (P) Serum FGF21 protein level measured by ELISA from mice in (K); *n* = 4–6. Data expressed as mean ± SEM. */^a^/^#^*p* < 0.05, **/^aa^/^##^*p* < 0.01, ***/^aaa^/^###^*p* < 0.001, ****/^aaaa^/^####^*p* < 0.0001 by one-way ANOVA (B), Student’s t test (D, G), Pearson correlation test (E), and two-way ANOVA (H, I, L, O, P). See also Figure S1.

In addition, pending food availability, behaviorally complex organisms will fast, sleep, do both, or do neither, throughout the circadian clock. To account for this, the hepatocyte harbors a cell-intrinsic core clock that includes: *Clock*, *Bmal1*, cryptochrome 1 (*Cry1*) and *Cry2*, and period homolog gene 1 (*Per1*) and *Per2*.^18-22^ These form a well-characterized transcriptional-translational feedback loop to maintain cyclical expression (Figure 1A).^23^ Genetic or environmental disruption of the core clock induces metabolic disease in mice and humans, while obesity alters rhythmic circadian gene expression.^24-27^ Recent data, however, suggest that the therapeutic metabolic response to time-restricted feeding is largely independent of the core clock.^28^ Thus, how and whether such signals interact to dictate fuel selection and survival remains incompletely addressed. Such dissonant findings led us to test the hypothesis that the hepatocyte integrates temporal and macronutrient sensing inputs to drive fuel selection.

Here, we identified hepatic *Per1* in multiple transcriptomic screens as a fasting-regulated, glucose-dependent factor in livers of fasting mice and in isolated hepatocytes.^29,30^ We show selective *Per1* induction during short- and long-term fasting, even in the absence of an intact core clock. Indirect calorimetry and *in vivo* heavy-isotope metabolic labeling further demonstrate that *Per1* drives the transition from glucose to fatty acid β-oxidation and manifold other fasting responses. Single-nucleus multiome sequencing and pharmacologic complementation experiments further demonstrate that *Per1* mediates substrate transition through altering hepatocyte subpopulation chromatin accessibility, acute transcriptional changes, and post-transcriptional control of *Fgf21,* which modulates pyruvate dehydrogenase kinase 4 (*Pdk4*) signaling during fasting to shunt carbon flux away from pyruvate dehydrogenase (PDH)-mediated glucose oxidation. Finally, we nominate composition-of-matter hepatocyte carbohydrate flux inhibitors, 4-trehelosamine (4-TA), 6-azido trehalose (6-TreAz), and IMCTA-C14 (IMCTA), as translatable means by which to selectively induce *Per1*. These findings identify hepatocyte *Per1* as a metabolic node that integrates temporal and nutritional input toward fuel selection and adaptation to shorter- and longer-term fasting.

## RESULTS

### Hepatocyte *Per1* mediates the adaptive metabolic response to fasting

*Per1* is a canonical circadian gene that is transcriptionally activated by *Bmal1* and *Clock* (Figure 1A). We first examined *Per1, Pgc1a,* and other core circadian gene expressions in liver in response to 12-h, 16-h, and 24-h starvation. This revealed significant induction of *Per1* and the canonical fasting-induced *Pgc1a* gene during both shorter-term and prolonged fasting (Figures 1B and S1A). In contrast, clock genes *Per2, Cry1, Cry2, Bmal1,* and *Clock* were not similarly induced and sustained throughout fasting for 12–24 h. We then defined how timing of fasting initiation and termination interacted with *Per1* induction in liver. We performed 16-h fasting in wild-type mice and harvested tissues every 4 h throughout the 24-h time course (Figure 1C). This revealed that fasting significantly induced *Per1* across all time points, although fasting periods predominantly encompassing the dark phase (e.g., fasting termination at zeitgeber time 0/24 [ZT0/24] and ZT4) had the greatest stimulating effect on *Per1* fasting induction (Figure 1D). Because mice primarily eat throughout the active/dark phase, we examined whether the magnitude of *Per1* induction simply correlated with the magnitude of food deficit. Correlation of food mass consumed in mice fed *ad libitum* and *Per1* induction during fasting at the same time point revealed a relationship between food deficit and *Per1* induction (Figure 1E). We next tested whether glucose transporter (GLUT) blockade is sufficient to induce *Per1* independent of full food withdrawal. We did this using trehalose-derived GLUT inhibitors trehalose, 4-TA, 6-TreAz, and IMCTA (Figure 1F).^31,32^ Each of these GLUT inhibitors induced *Per1* up to 3.5-fold, without inducing *Per2,* in isolated primary hepatocytes (Figure 1G). Consistent with this, we examined the dose responsiveness of hepatocytes to glucose withdrawal. We treated murine AML12 hepatocytes with 0–25 mM glucose in regular growth medium and quantified *Per1* and *Per2* gene expression. *Per1* but not *Per2* increased in response to deceasing glucose exposure (Figure S1B). Again, this indicated that glucose withdrawal is sufficient to induce hepatocyte *Per1* and that *Per1* regulation in response to glucose withdrawal is distinct from *Per2* regulation.

To define broader *Per1* functions during starvation, we quantified the effect of hepatocyte *Per1* knockdown on autophagic flux and fasting signaling *in vitro.* We modulated *Per1* expression by adenovirus-driven short hairpin RNA (Ad-shPer1) in AML12 and starved the cells in nutrient-depleted medium (Figures 1H and S1C). We then quantified LC3B-II accumulation as a biomarker of autophagic flux, a process that is activated in liver during fasting. *Per1* knockdown attenuated starvation-induced LC3B-II accumulation in hepatocytes treated with adenovirus encoding *Per1* short hairpin RNA when compared with GFP-expressing hepatocytes (Figure 1I). This occurred in the context of increased phosphorylation of the autophagy-inhibiting mammalian target of rapamycin (mTOR) complex 1 target site, pULK1^Ser757^, in fasting *Per1*-deficient hepatocytes (Figure 1I).^33^

This prompted us to generate mice harboring homozygous floxed hepatocyte-specific *Per1* alleles using CRISPR-Cas9-mediated gene editing. We inserted *LoxP* sites flanking exons 4 and 10 of *Per1* (Figure 1J) and treated these mice with adeno-associated virus serotype 8 (AAV8)-encoding Cre recombinase under thyroxine-binding globulin promoter control (AAV8-TBG-Cre) to delete *Per1* specifically in hepatocytes (*Per1*^iLKO^, Figure 1K). 2 weeks after AAV8 treatment, we subjected these mice to 16-h fasting or fasting with refeeding (Figure 1K). Fasting *Per1*^iLKO^ mice exhibited impaired fatty acid, ketone body, and intrahepatic triglyceride (TG) accumulation, and this was also confirmed by oil red O (ORO) staining (Figures 1L and 1M). No significant difference in body-weight change, serum glucose, and liver free fatty acid level was observed between different genotypes (Figures S1D and S1E).

Bulk transcriptomics in refed and fasting *Per1*^fl/fl^ and *Per1*^iLKO^ liver allowed us to identify potential pathways underlying defective substrate selection in *Per1*^iLKO^ mice. Unsupervised clustering demonstrated greater separation between fed and fasting *Per1*^fl/fl^ liver transcriptome versus *Per1*^iLKO^ livers under the same conditions (Figure 1N). Transcriptional pathways upregulated included mTOR and insulin signaling, whereas downstream peroxisome proliferator-activated receptor signaling and fatty acid oxidation were downregulated in fasting *Per1*^iLKO^ versus *Per1*^fl/fl^ fasted liver (Figure S1F). Among differentially expressed genes (DEGs) were the genes encoding both the fasting-induced hepatokine *Fgf21* and *Pdk4* (Figure 1N). We verified deletion of hepatocyte *Per1* in *Per1*^iLKO^ mice and validated *Fgf21* and *Pdk4* gene expression and *Fgf21* peptide defects by quantitative real-time PCR (real-time qPCR) (Figure 1O) and ELISA (Figure 1P). This confirmed impaired fasting-induced hepatic *Fgf21* and *Pdk4* expression and impaired fasting-induced FGF21 peptide in *Per1*^iLKO^ liver and serum, respectively (Figures 1O and 1P). Given that *Fgf21* and *Pdk4* are both regulated by *Ppara*,^34,35^ we measured *Ppara* expression along with its downstream target genes (Figure S1G). Fasting significantly induced *Ppara* and several target genes in both *Per1*^fl/fl^ and *Per1*^iLKO^ liver (Figure S1G). Together, the data indicate that *Per1* regulates *Fgf21* and *Pdk4* without major contribution by the *Ppara* pathway.

### Hepatocyte *Per1* mediates transcriptional changes and chromatin remodeling in a fasting-responsive hepatocyte subpopulation

**Figure 2.**
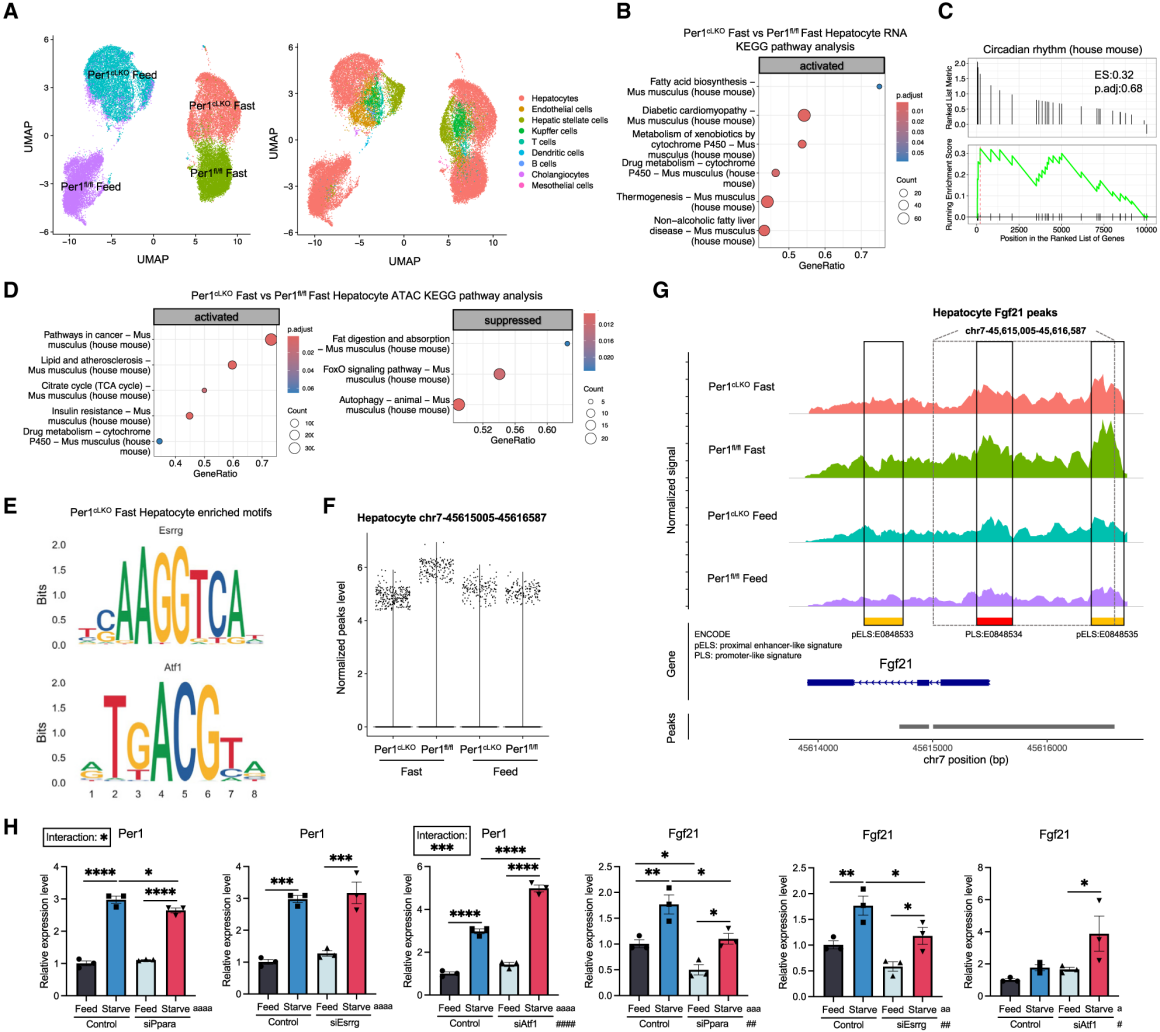
Single-nucleus multiome sequencing reveals that *Per1* drives chromatin remodeling in a hepatocyte subpopulation (A) Uniform manifold approximation and projection for dimension reduction (UMAP) plot on single-nucleus multiome sequencing analysis of liver tissues from *Per1*^fl/fl^ and *Per1*^cLKO^ (*Per1*^fl/fl^, Alb-Cre) fed *ad libitum* or fasted for 16 h (left). Nine major cell types were identified (right). In total, 9,871 (*Per1*^fl/fl^ Feed), 8,702 (*Per1*^fl/fl^ Fast), 9,494 (*Per1*^cLKO^ Feed), and 8,731 (*Per1*^cLKO^ Fast) nuclei were analyzed in each condition; *n* = 3. (B) Kyoto Encyclopedia of Genes and Genomes (KEGG) pathway analysis of differentially expressed genes (DEGs) between hepatocyte populations from *Per1*^cLKO^ Fast group and *Per1*^fl/fl^ Fast group. (C) Gene set enrichment analysis of circadian rhythm pathway between DEGs from hepatocytes in *Per1*^cLKO^ Fast group and *Per1*^fl/fl^ Fast group. (D) KEGG pathway analysis of differentially expressed peak signals between hepatocyte population from *Per1*^cLKO^ Fast group and *Per1*^fl/fl^ Fast group. (E) Enriched motifs identified in hepatocyte population from *Per1*^cLKO^ Fast group. (F) Quantification of normalized peak level at region chr7-45615005-45616587 in hepatocyte population from each group. In total, 9,267 (*Per1*^fl/fl^ Feed), 7,626 (*Per1*^fl/fl^ Fast), 5,445 (*Per1*^cLKO^ Feed), and 5,525 (*Per1*^cLKO^ Fast) nuclei were identified as hepatocytes and analyzed in each condition; *n* = 3. (G) Coverage plot at *Fgf21* locus from hepatocyte population. Functional units were identified based on the Encyclopedia of DNA Elements (ENCODE) database. (H) Relative expression of *Per1* and *Fgf21* in AML12 cells treated with siRNA targeting *Ppara, Esrrg*, or *Atf1* followed by medium change to complete or starvation medium; *n* = 3. Data expressed as mean ± SEM. */^a^/^#^*p* < 0.05, **/^aa^/^##^*p* < 0.01, ***/^aaa^/^###^*p* < 0.001, ****/^aaaa^/^####^*p* < 0.0001 by two-way ANOVA (H). See also Figure S2 and Table S1.

To gain deeper insight into hepatocyte-intrinsic defects during fasting in *Per1*^fl/fl^ or *Per1*^fl/fl^;Alb-Cre (*Per1*^cLKO^) liver, we lever-aged single-nucleus multiome sequencing (e.g., single-nucleus RNA [snRNA] sequencing and single-nucleus assay for transposase-accessible chromatin [snATAC] sequencing) to define cellular subpopulations regulated by hepatocyte *Per1* at the levels of RNA and chromatin (Figure 2A). We utilized a stable germline hepatocyte-specific targeting approach here to minimize acute gene expression and chromatin changes that might be observed due to an AAV8-mediated TBG-promoter-driven Cre targeting strategy. In this model, uniform manifold approximation and projection for dimension reduction (UMAP) plot-integrated snRNA-seq and snATAC-seq data revealed sharp demarcation between clusters of samples from each treatment and genotype (Figure 2A). Cell-marker analysis verified identification of a broad complement of liver cells, including hepatocyte, endothelial, stellate, Kupffer cells, T cells, dendritic cells, B cells, cholangiocytes, and mesothelium cells (Figures S2A-S2C). Expression of canonical hepatocyte genes *Alb, Cyp7b1, Mug1, Cyp4a14, Egfr,* and *Saa1* defined the hepatocyte population in all groups (Figures S2B and S2C). We did not detect transcriptomic signal of *Fgf21* from our hepatocyte population in the single-nucleus multiome data. No prior groups, to our knowledge, reported snRNA-seq *Fgf21* expression in hepatocyte population.^36^ Upregulation of *Ppara* and *Pck1* expression in fasting *Per1*^fl/fl^ mice when compared with fed *Per1*^fl/fl^ mice internally validated our fasting and analytical approaches (Figure S2D). Kyoto Encyclopedia of Genes and Genomes (KEGG) pathway analysis comparing *Per1*^fl/fl^ and *Per1*^cLKO^ fasted hepatocytes revealed activation of genes involved in lipid deposition in the *Per1*^cLKO^ fasted hepatocytes, without any detectable defect in the circadian rhythm pathway (Figures 2B and 2C). snATAC-seq revealed enhanced chromatin accessibility in cancer, lipid, TCA-cycle, and insulin-resistance pathways in fasted *Per1*^cLKO^ hepatocytes. In addition, this analysis revealed suppressed chromatin accessibility in fat-metabolism- and autophagy-related pathways in fasted *Per1*^cLKO^ hepatocytes (Figure 2D), which were oppositely regulated in *Per1*^fl/fl^ fasted hepatocytes when compared to *Per1*^fl/fl^ fed hepatocytes (Figures S2E and S2F).

Motif analysis demonstrated a significant enrichment in *Esrrg* and *Atf1* transcription factors in *Per1*^cLKO^ fasted hepatocytes (Figure 2E). In light of the fact that *Esrrg* is a known transcriptional activator that directly binds to the *Fgf21* promoter,^37^ we interrogated hepatocyte chromatin accessibility near the *Fgf21* locus along chromosome 7 and made at least two key observations. First, fasting-induced chromatin accessibility at *Fgf21,* but not *Pdk4,* locus was reduced in *Per1*^cLKO^ hepatocytes during fasting at the promoter and enhancer regions (Figures 2G and S2G). Overall, the data indicated cell subpopulation-intrinsic regulation of *Fgf21* transcription. We therefore directly examined *Ppara, Esrrg,* and *Atf1* function in *Per1-Fgf21* expression in fasting AML12 hepatocytes using a genetic loss-of-function experimental approach. This revealed that small interfering RNA (siRNA)-mediated *Ppara* and *Esrrg* knockdown impaired maximal *Fgf21* induction during starvation (Figures 2H and S2H). Although no motif was significantly enriched in *Per1*^fl/fl^ fasted hepatocytes, *Esrrg* was also enriched in hepatocytes from fed *Per1*^cLKO^ mice (Table S1). Direct functional and multiomic data together indicate that *Per1* may regulate an *Esrrg-Fgf21* axis.

**Figure 3.**
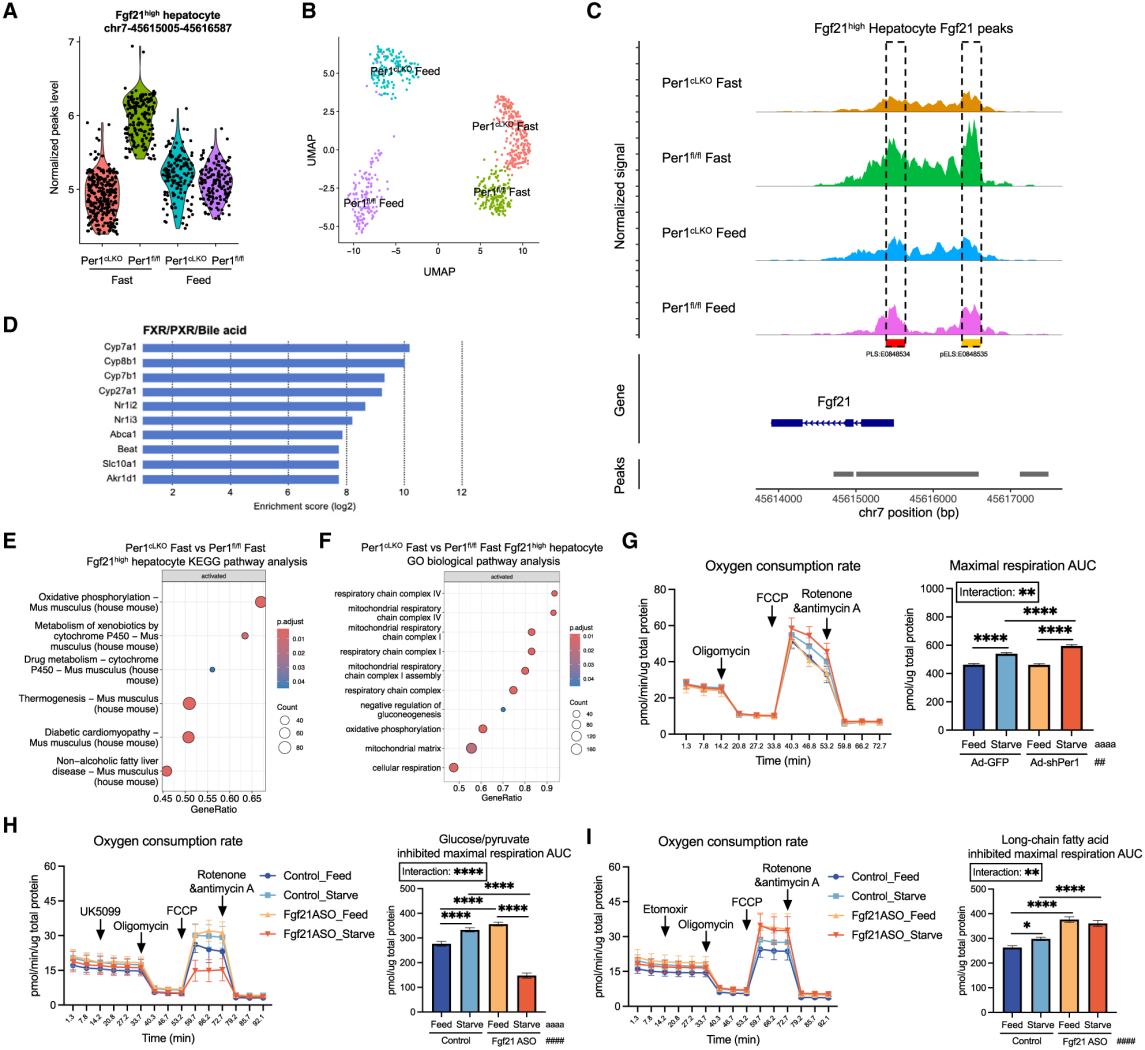
Hepatocyte subpopulation analysis demonstrates enhanced glucose oxidation in fasting *Per1*^LKO^ hepatocytes (A) Hepatocyte subpopulation selection based on the normalized peak signal at chr7-45615005-45616587 region. Quantification of the normalized peak signal in each group. In total, of 144 (*Per1*^fl/fl^ Feed), 165 (*Per1*^fl/fl^ Fast), 145 (*Per1*^cLKO^ Feed), and 253 (*Per1*^cLKO^ Fast) nuclei were identified as *Fgf21*^high^ hepatocyte subpopulation and analyzed in each condition; *n* = 3. (B) Selected hepatocyte subpopulation distribution in the UMAP based on the treatment. (C) Coverage plot at *Fgf21* locus from selected hepatocyte subpopulation (*Fgf21*^high^) and their corresponding *Per1* expression level. (D) Farnesoid X receptor (FXR)/pregnane X receptor (PXR)/bile acid pathway analysis from Comprehensive Multi-omics Platform for Biological Interpretation (COMPBIO) from differentially regulated genes in *Fgf21*^high^ hepatocyte subpopulation from *Per1*^cLKO^ Fast group and *Per1*^fl/fl^ Fast group. (E) KEGG pathway analysis of DEGs from *Fgf21*^high^ hepatocyte subpopulation between *Per1*^cLKO^ Fast group and *Per1*^fl/fl^ Fast group. (F) Gene ontology (GO) pathway analysis of DEGs from *Fgf21*^high^ hepatocyte subpopulation between *Per1*_iLKO_ Fast group and *Per1*^fl/fl^ Fast group. (G) Seahorse XF Cell Mito Stress analysis of AML12 cells transduced with Ad-GFP or Ad-shPer1 for 48 h followed by medium change to either complete or starvation medium for 6 h (left), and area under the curve (AUC) quantification of the maximal respiration rate (right); *n* = 12. (H) Seahorse XF glucose/pyruvate oxidation stress analysis of AML12 cells treated with *Fgf21* antisense oligonucleotide (ASO) for 48 h followed by medium change to either complete or starvation medium for 6 h (left), and AUC quantification of the inhibited maximal respiration rate (right); *n* = 12. (I) Seahorse XF long-chain fatty acid oxidation stress analysis of AML12 cells treated with *Fgf21* antisense oligonucleotide (ASO) for 48 h followed by medium change to either complete or starvation medium for 6 h (left), and AUC quantification of the inhibited maximal respiration rate (right); *n* = 12. Data expressed as mean ± SEM. */^a^/^#^*p* < 0.05, **/^aa^/^##^*p* < 0.01, ***/^aaa^/^###^*p* < 0.001, ****/^aaaa^/^####^*p* < 0.0001 by two-way ANOVA (G–I). See also Figure S2 and Table S2.

Second, we noted a subpopulation of hepatocytes that responded and maintained *Fgf21* chromatin accessibility, as characterized by a normalized peak score >4 (e.g., *Fgf21*^high^ hepatocytes, Figures 2F, 2G, and 3A-3C). These *Fgf21*^high^ hepatocytes comprised a relatively modest proportion of the overall hepatocyte population: *Per1*^fl/fl^ Feed, 1.6%; *Per1*^fl/fl^ Fast, 2.1%; *Per1*^cLKO^ Feed, 2.6%; *Per1*^cLKO^ Fast, 4.5%. The *Fgf21*^high^ hepatocyte subpopulation was dynamic and responded to fasting by increasing chromatin accessibility at the *Fgf21* locus in fasting *Per1*^fl/fl^ mice but not in fasting *Per1*^cLKO^ mice (Figures 3A-3C). Isolated subpopulation analysis of the *Fgf21*^high^ hepatocyte transcriptomic profile further revealed enrichment of genes involved in the bile acid/farnesoid X receptor pathways in fasting *Per1*^cLKO^ and *Fgf21*^high^ hepatocytes (Figures 3D, S2E, and S2F; Table S2).

We next asked whether zonal distribution could at least in part characterize the *Fgf21*^high^ hepatocyte population. This revealed significant enrichment and a trend toward significant enrichment of central vein zonal marker expression *Cyp2e1* and *Glul,* respectively in the *Fgf21*^high^ population (Figure S2I). This suggested potential zonal predilection associated with fasting sensitivity at the *Fgf21* locus.^38^ Oxidative phosphorylation, thermogenic, and respiration pathways were further enriched in *Per1*^cLKO^ *Fgf21*^high^ hepatocytes that failed chromatin fasting induction (Figures 3E and 3F). Again, no defect in chromatin accessibility was found at the *Pdk4* locus (Figure S2J), and this correlated with *in vitro* enhancement in mitochondrial respiration in starved *Per1*-deficient AML12 hepatocytes subjected to Seahorse mitochondrial respirometry (Figure 3G).

In a distinct line of mitochondrial respiration experiments, we next sought to define the effect of inhibiting fatty acid or glucose/pyruvate oxidation in hepatocytes with or without *Fgf21* knockdown. We measured real-time mitochondrial respiration in the presence or absence of pyruvate carrier inhibitor (UK5099) or fatty acid oxidation inhibitor (etomoxir) in AML12 hepatocytes with or without *Fgf21* antisense oligonucleotide (Figure S2K). Cells lacking *Fgf21* exhibited significantly reduced mitochondrial respiration when pyruvate oxidation was inhibited. In contrast, inhibiting fatty acid oxidation in fasting *Fgf21-*deficient hepatocytes had no significant impact on maximal respiration when compared to fed *Fgf21*-deficient hepatocytes (Figures 3H and 3I). This indicated that the absence of *Fgf21* in hepatocytes causes greater reliance on glucose/pyruvate than fatty acid as their major energy source during fasting. Together, the data indicate a specific hepatocyte-intrinsic subpopulation of fasting non-responsive hepatocytes that is demarcated by failed *Fgf21* accessibility, upregulated bile acid signaling, and increased oxidative phosphorylation.

### The hepatocyte-intrinsic circadian clock is dispensable for fasting-induced *Per1* regulation

**Figure 4.**
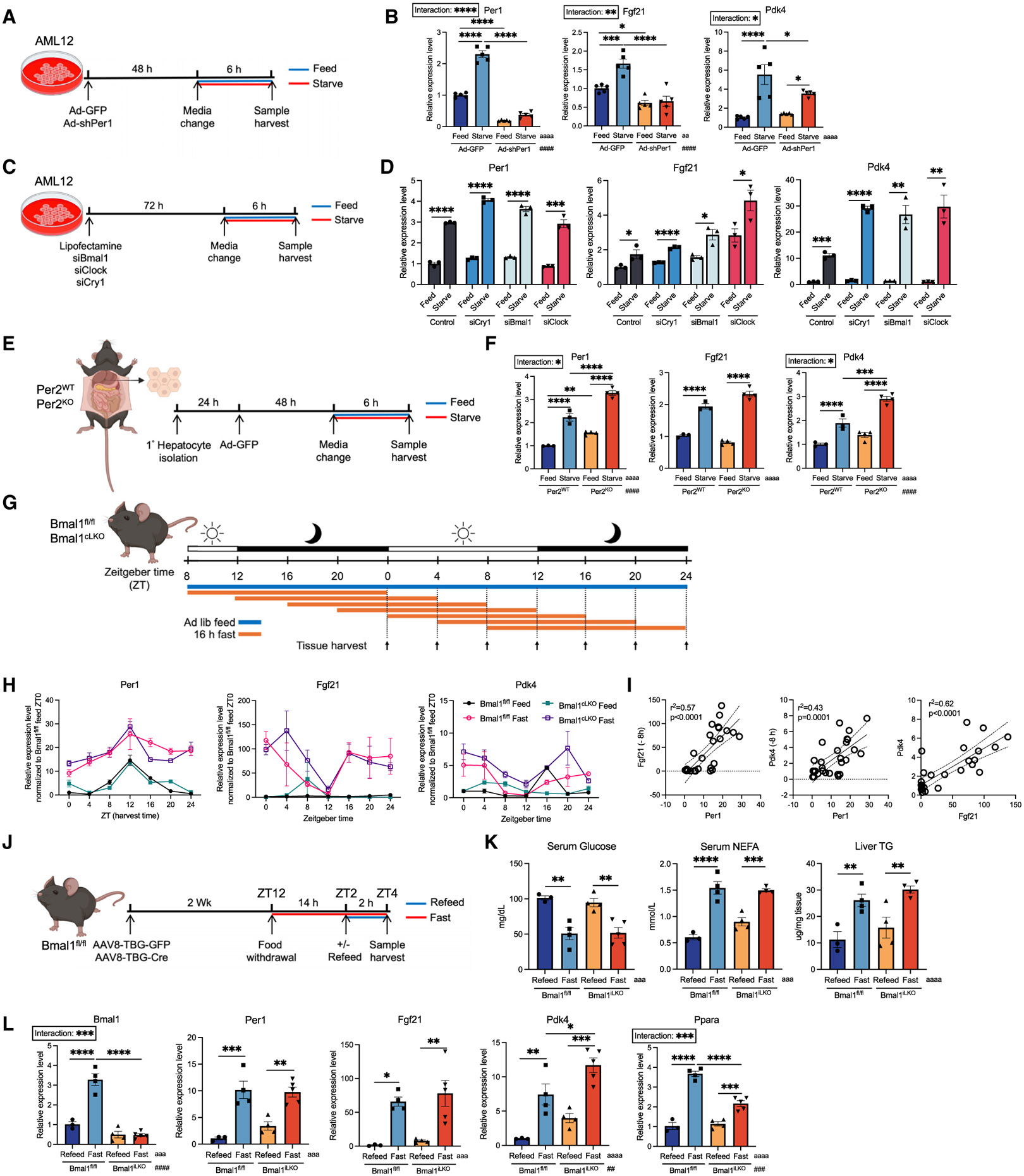
Fasting induces *Per1, Fgf21*, and *Pdk4* independent of the core circadian clock (A and C) Schematic of *in vitro* feed/starve experiment in AML12 cells treated with either adenovirus or siRNA targeting *Per1, Bmal1, Clock*, or *Cry1*. (B) Relative expression level of *Per1, Fgf21*, and *Pdk4* in AML12 cells treated with Ad-GFP or Ad-sh*Per1*, fed or starved; *n* = 5. (D) Relative expression level of *Per1, Fgf21*, and *Pdk4* in AML12 cells treated with lipofectamine (Control), si*Bmal1*, si*Clock*, or si*Cry1*, fed or starved; *n* = 3. (E) Schematic of *ex vivo* primary hepatocyte starvation experiment from *Per2*^WT^ and *Per2*^KO^ female mice. (F) Relative expression level of *Per1, Fgf21*, and *Pdk4* in primary hepatocytes isolated from *Per2*^WT^ or *Per2*^KO^ mice, fed or starved; *n* = 3–4. (G) Schematic of *in vivo* time-course experiment in *Bmal1*^fl/fl^ and *Bmal1*^cLKO^ (*Bmal1*^fl/fl^, Alb-Cre) mice. Mice were either fed *ad libitum* or fasted for 16 h, and liver tissues were harvested every 4 h in a 24-h duration; *n* = 3–4. (H) Relative expression level of *Per1, Fgf21*, and *Pdk4* (normalized to ZT0 *Bmal1*^fl/fl^ feed) from liver in (G). (I) Correlation test result between normalized expression level of *Per1* and *Fgf21* (−8 h or +16 h) (left), *Per1* and *Pdk4* (−8 h or + 16 h) (middle), and *Fgf21* and *Pdk4* (right) in (H). Dotted line denotes the 95% confidence interval for the simple linear regression calculation. (J) Schematic of the 14 h + 2 h fast/refeed experimental design in *Bmal1*^fl/fl^ (*Bmal1*^fl/fl^, AAV8-TBG-GFP) and *Bmal1*^iLKO^ (*Per1*^fl/fl^, AAV8-TBG-Cre) mice; *n* = 3–5. (K) Serum glucose (left), NEFA (middle), and hepatic TG (right) level from mice in (J). (L) Relative expression level of liver *Bmal1, Per1, Fgf21, Pdk4*, and *Ppara* from mice in (J). Data expressed as mean ± SEM. */^a^/^#^*p* < 0.05, **/^aa^/^##^*p* < 0.01, ***/^aaa^/^###^*p* < 0.001, ****/^aaaa^/^####^*p* < 0.0001 by two-way ANOVA (B, F, K, L), Student’s t test (D), and Pearson correlation test (I). See also Figures S3 and S4.

*Per1* canonically functions in concert with other core circadian genes, including *Per2, Cry1/2, Bmal1*, and *Clock*.^39,40^ We tested the extent to which *Per1* regulatory effects on fasting require circadian regulation. First, *Per1* knockdown followed by low-glucose, no-serum medium exposure in AML12 cells revealed that *Per1* knockdown reduced *Fgf21* and *Pdk4* gene induction in response to nutrient withdrawal (Figures 4A and 4B). In contrast, real-time qPCR confirmed intact transcriptional activation of *Per1, Fgf21,* and *Pdk4* during starvation in cells deficient for any of the core clock genes-*Cry1, Bmal1*, and *Clock,* whereas several other circadian genes were disrupted when knocking down these core circadian genes. (Figures 4C, 4D, and S3A-S3D). Similarly, we challenged *ex vivo* primary hepatocytes isolated from *Per2*^WT^ and *Per2*^KO^ mice with starvation medium (Figure 4E), and *Per2*^KO^ hepatocytes exhibited intact *Per1, Fgf21,* and *Pdk4* induction during starvation when compared with Per2^WT^ hepatocytes (Figure 4F).

To define circadian dependence of the *Per1-Fgf21* pathway *in vivo*, we examined *Per1* activation during fasting throughout the circadian clock in hepatocyte-specific *Bmal1*-deficient mice (Figure 4G). We selected *Bmal1*^cLKO^ (*Bmal1*^fl/fl^, Alb-Cre) mice as our model, because *Bmal1* is required for diurnal behaviors in mice and rhythmic expression of *Per1/2*.^39,41^ We withdrew food every 4 h or fed mice *ad libitum* in *Bmal1*^fl/fl^ or *Bmal1*^cLKO^ mice (Figure 4G). Mice were sacrificed 16 h after food withdrawal throughout ZT0–ZT24. Strikingly, *Per1* expression maintained its rhythmicity and was upregulated in 16-h-fasted livers, independent of *Bmal1* and independent of fasting initiation timing (Figure 4H). In contrast, *Per2* completely lost rhythmicity in the absence of hepatocyte *Bmal1* and was again minimally responsive to fasting (Figure S3E). Expression of other core circadian genes also depended on *Bmal1* (Figure S3E). Moreover, *Fgf21* and *Pdk4* expression was induced in *Bmal1*^fl/fl^ mice during fasting at most time points and was activated in *Bmal1*^cLKO^ mice independent of fasting initiation timing (Figure 4H). We noted that *Per1-Fgf21-Pdk4* input response remained a significant linear stimulus-response relationship at most time points throughout the time course (Figure S3F). However, we observed a strong correlation between *Per1* and downstream *Fgf21-Pdk4* expression 8 h later to yield the strongest correlation coefficient (Figure 4I). No time lag was required to account for *Fgf21* and *Pdk4* alignment (Figures 4I and S3F). The data indicate a *Per1-Fgf21-Pdk4* association during fasting, consistent with the possibility that gene expression and chromatin remodeling constitute a key aspect of the *Per1*-mediated fasting response. We also noted that in the standard fast/refeed model, *Bmal1*^iLKO^ (*Bmal1*^fl/fl^, AAV8-TBG-Cre) maintained intact serum glucose, serum non-esterified fatty acid, and intrahepatic liver TG as well as intact *Per1*, *Fgf21,* and *Pdk4* gene expression during fasting when compared with fed mice (Figures 4J-4L). Surprisingly, we identified a significant defect in *Ppara* expression in fasting mice lacking hepatocyte *Bmal1,* further suggesting a *Ppara*-independent activation of *Fgf21-Pdk4* in fasting liver (Figure 4L).

We next sought to better understand temporal and nutritional interactions in male *Per1*^fl/fl^ and *Per1*^iLKO^ mice. We therefore performed a similar fast/feed time course in *Per1*^fl/fl^ and *Per1*^iLKO^ mice (Figure S4A). This again revealed that *Fgf21* and *Pdk4* gene expression were activated during fasting in *Per1*^fl/fl^ mice. *Fgf21* and *Pdk4* were significantly reduced in *Per1*^iLKO^ mice subjected to fasting that spans the entire dark phase (Figures S4B and S4C). That is, 16-h-fasted *Per1*^iLKO^ mice analyzed at ZT0/24 and ZT4 exhibited the greatest magnitude of *Fgf21-Pdk4* gene expression defect when compared with fasting *Per1*^fl/fl^ mice (Figures 1D, S4B, and S4C). To then test the extent to which *Per1* regulates *Fgf21* and *Pdk4* in female mice, we performed a 16-h fasting experiment in female *Per1*^fl/fl^ mice treated with AAV8-TBG-GFP or AAV8-TBG-Cre (Figure S4D). We showed that *Per1* was significantly induced by fasting in control mice at both ZT0/24 and ZT4, and *Fgf21* and *Pdk4* were significantly reduced in fasting *Per1*^iLKO^ mice at the same time points (Figure S4E).

### Pyruvate dehydrogenase links *Per1* to proper fuel selection during fasting/feeding

**Figure 5.**
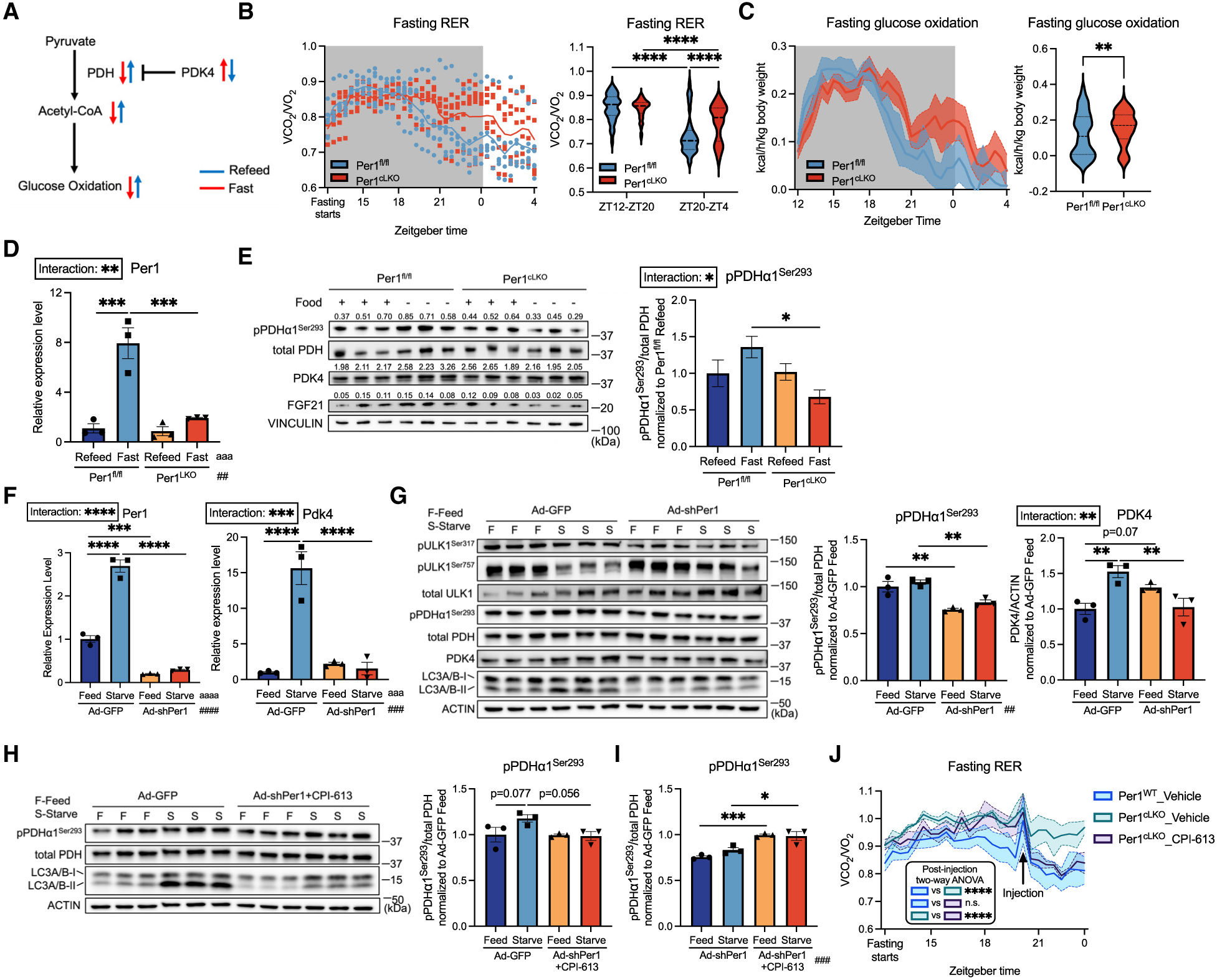
PDH inhibition restores substrate predilection from glucose to fatty acid in *Per1*^LKO^-fasted mice (A) Schematic of the fasting-induced *Pdk4*-mediated inhibition of glucose oxidation. (B) Respiratory exchange ratio (RER) measured during 16 h of fasting in *Per1*^fl/fl^ and *Per1*^cLKO^ (*Per1*^fl/fl^, Alb-Cre) mice in indirect calorimetry (left), and quantification of RER during fasting from ZT12 to ZT20 and ZT20 to ZT4 (right); *n* = 6. (C) Glucose oxidation rate calculated based on VCO_2_ and VO_2_ during fasting. Shade denotes SEM (left) and quantification (right) from mice in (B); *n* = 6. (D) Relative expression level of *Per1* in liver from *Per1*^fl/fl^ or *Per1*^cLKO^ mice underwent 14 h + 2 h fast/refeed; *n* = 3. (E) Immunoblot analysis of liver tissues from (D), quantification labeled on the top of each band (pPDHa1^Ser293^ was normalized to total PDH) (left), and quantification of pPDHa1^Ser293^ level normalized to *Per1*^fl/fl^ refeed (right); *n* = 3. (F) Relative expression level of *Per1* and *Pdk4* from AML12 cells transduced with Ad-GFP or Ad-sh*Per1* for 48 h followed by medium change to either complete or starvation medium for 48 h; *n* = 3. (G) Immunoblot analysis of AML12 cells as described in (F) (left), and quantification of pPDHa1^Ser293^ and PDK4 (right); *n* = 3. (H) Immunoblot analysis of AML12 cells as described in (F), with additional treatment of 25 μM CPI-613 2 h prior to harvest in Ad-sh*Per1*-treated cells (left). Quantification of pPDHα1^Ser293^ (right); *n* = 3. (I) pPDHa1^Ser293^ quantification from sh*Per1*-treated AML12 cells with or without CPI-613 from (G) and (H). pPDHa1^Ser293^ level was normalized to corresponding Ad-GFP-treated cells cultured in complete medium; *n* = 3. (J) RER measured during 16 h of fasting in *Per1*^fl/fl^ and *Per1*^cLKO^ mice in indirect calorimetry injected with either vehicle or 25 mg/kg CPI-613 at ZT20; *n* = 3. Data expressed as mean ± SEM. */^a^/^#^*p* < 0.05, **/^aa^/^##^*p* < 0.01, ***/^aaa^/^###^*p* < 0.001, ****/^aaaa^/^####^*p* < 0.0001 by two-way ANOVA (B, D–J) and Student’s t test (C). See also Figure S5.

*Pdk4* regulates fuel selection from glucose to fatty acid during fasting through phosphorylating and inhibiting PDH activity (Figure 5A).^42^ Defective *Pdk4* transcriptional activation and fatty acid oxidation in *Per1*^iLKO^ fasted liver prompted the hypothesis that hepatocyte *Per1*-deficient mice exhibit impaired fuel utilization from glucose to fatty acid oxidation during fasting. To test this, we quantified substrate selection *in vivo* by indirect calorimetry. *Per1*^fl/fl^ mice appropriately decreased glucose oxidative capacity during fasting, as indicated by a decrease in respiratory exchange ratio (RER). In contrast, *Per1*^cLKO^ mice exhibited an attenuated RER reduction (Figures 5B and 5C) accompanied by a modestly higher hepatic glycogen utilization (Figure S5A). Similarly, *Per1*^cLKO^ mice failed to completely suppress heat generation, oxygen uptake (VO_2_), and carbon dioxide output (VCO_2_) when compared with *Per1*^fl/fl^ mice during fasting. However, we observed no genotype-driven differences in food consumption, total activity, total body weight prior to fasting, or total activity during fasting (Figures S5B-S5F).

We then asked whether impaired fuel switching and enhanced glucose oxidation during fasting were due to failed inhibition of PDH. We measured liver pPDHα1^Ser293^ level in *Per1*^fl/fl^ and *Per1*^cLKO^ mice after 14 h + 2 h fast/refeed. *Per1*^cLKO^ mice exhibited an increase in hepatic pPDHα1^Ser293^ during refeeding and a significant reduction in pPDHα1^Ser293^ during fasting (Figures 5D and 5E). Again, we saw the defective activation in FGF21 protein in *Per1*^cLKO^ fasted liver (Figure 5E). We subjected AML12 cells *in vitro* to 48 h of starvation and demonstrated impaired autophagic flux and decreased starvation-induced PDH phosphorylation, PDK4 protein, and gene expression (Figures 5F and 5G), suggesting that *Per1* drives the hepatocyte-intrinsic PDK4-PDH regulation.

Following the observation of a defective pPDHα1^Ser293^ level and abnormal glucose oxidation during fasting, we tested whether pharmacologically inhibiting PDH activity using CPI-613 (devimistat)^43^ is sufficient to reverse dysregulated *Per1*^cLKO^ fasting glucose metabolism. Previously, CPI-613 treatment was shown to significantly increase the pPDH^Ser293^ level in leukemia K562 cell line.^44^ We also verified that CPI-613 treatment significantly increased the level of pPDH^Ser293^ in sh*Per1*-treated AML12 cells (Figures 5H and 5I). *In vivo* injection of CPI-613 at ZT20 also reversed the inappropriately elevated RER observed in *Per1*^cLKO^ mice during fasting (Figure 5J). In summary, we identified impaired substrate flexibility in fasting *Per1*^cLKO^ mice, and this was associated with reduced *Pdk4* expression and lower pPDH^Ser293^ phosphorylation. Pharmacological PDH inhibition is sufficient to reverse the loss of substrate flexibility in *Per1*^cLKO^ mice.

### Hepatocyte *Fgf21* links *Per1* to *Pdk4*-mediated fasting glucose oxidation attenuation

**Figure 6.**
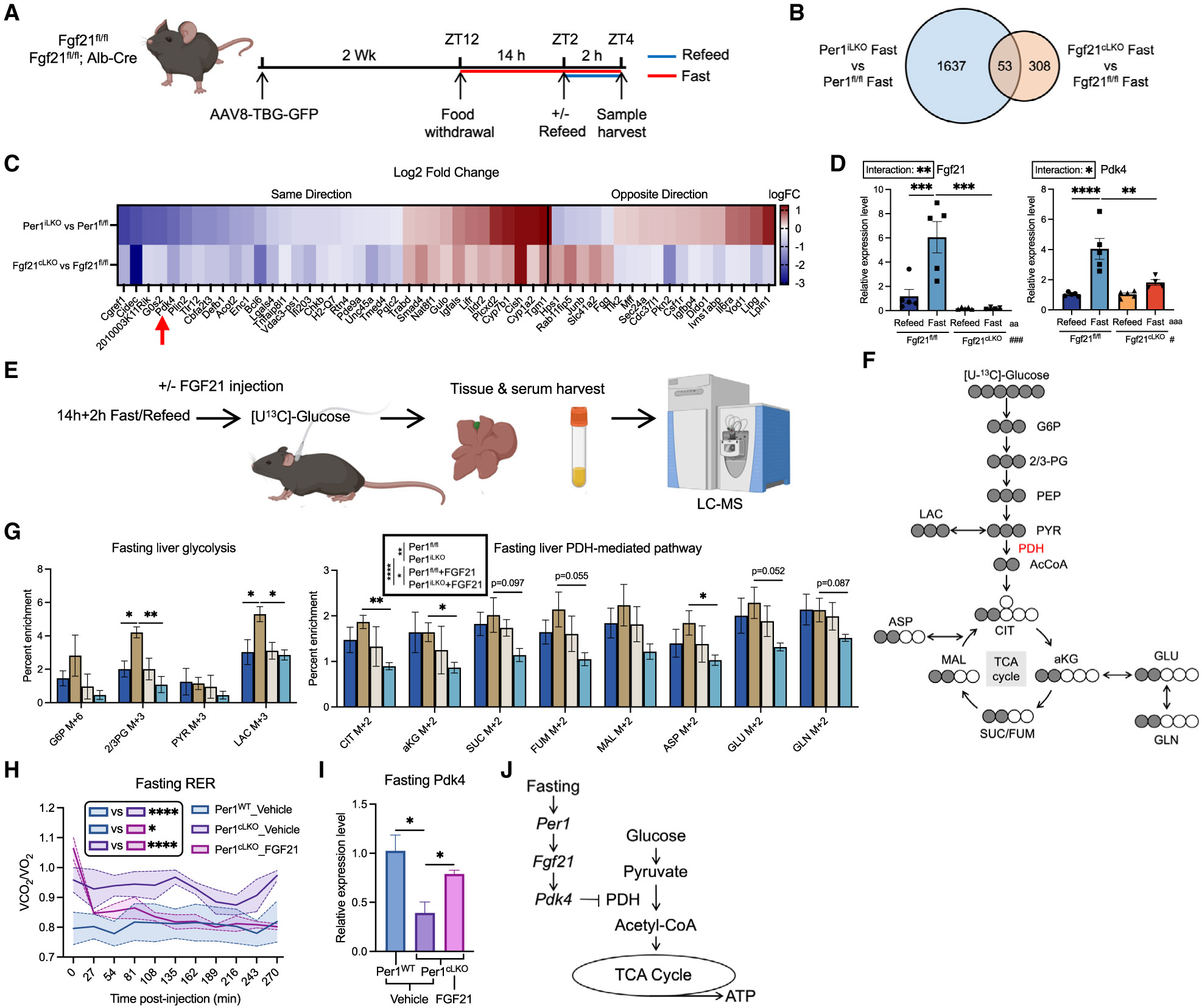
FGF21 drives the *Pdk4*-mediated inhibition of glucose oxidation during fasting (A) Schematic of the 14 h + 2 h fast/refeed experimental design in *Fgf21*^fl/fl^ and *Fgf21*^cLKO^ (*Fgf21*^fl/fl^, Alb-Cre) mice. (B) Diagram of differentially regulated gene (*p* < 0.05) distribution in fasted liver found in *Per1*^iLKO^ and *Fgf21*^cLKO^ mice compared to corresponding floxed control fasted mice; *n* = 3. (C) Heatmap of the 53 overlapped genes found in (B) and their log fold change (logFC). (D) Relative expression level of *Fgf21* and *Pdk4* in liver harvested from (A); *n* = 4–5. (E) Schematic of *in vivo* tracing experiment. In brief, [U-^13^C]glucose was infused to *Per1*^fl/fl^ or *Per1*^iLKO^ mice that underwent 14 h + 2 h fast/refeed, with or without injection of 1 mg/kg FGF21; *n* = 3. (F) Schematic of ^13^C-labeled (gray) and unlabeled carbon (white) distribution from [U-^13^C]glucose in glycolysis and PDH-mediated tricarboxylic acid (TCA) cycle. (G) Hepatic percent enrichment of correspondingly labeled metabolites involved in glycolysis and PDH-mediated TCA cycle in fasted mice from (E); *n* = 3. (H) RER measured in *Per1*^WT^ and *Per1*^cLKO^ (*Per1*^fl/fl^, Alb-Cre) mice injected with either vehicle or mouse FGF21 recombinant protein (1 mg/kg) after 8 h of fasting (fasting started at ZT12); *n* = 3. (I) Relative expression level of *Pdk4* in liver harvested from (H); *n* = 3. (J) Schematic of the regulatory pathway *Per1-Fgf21-Pdk4*-PDH during fasting. Data expressed as mean ± SEM. */^a^/^#^*p* < 0.05, **/^aa^/^##^*p* < 0.01, ***/^aaa^/^###^*p* < 0.001, ****/^aaaa^/^####^*p* < 0.0001 by two-way ANOVA (D, G) and Student’s t test (I). See also Figure S6.

The strong correlation between *Fgf21* and *Pdk4* expression led us to quantify the degree to which fasting mice lacking hepatocyte-specific *Fgf21* also exhibit aspects of impaired fasting signaling, as we observed in *Per1*^LKO^ mice. We performed 14 h + 2 h fast/refeed in *Fgf21*^fl/fl^ and *Fgf21*^cLKO^ (*Fgf21*^fl/fl^, Alb-Cre) mice (Figure 6A). Fasting *Fgf21*^cLKO^ mice upregulated *Per1* to the same degree as in fasting *Fgf21*^fl/fl^ mice (Figure S6A). Bulk RNA sequencing revealed 308 DEGs in liver from fasting *Fgf21*^cLKO^ mice versus *Fgf21*^fl/fl^ mice. Among them, 53 DEGs were differentially expressed when comparing *Per1*^iLKO^ fasted and *Per1*^fl/fl^ fasted mice. Thirty-five of 53 DEGs, including *Pdk4,* were similarly altered in the same direction in fasting *Per1*^iLKO^ and fasting *Fgf21*^cLKO^ versus fasting floxed control mice (Figures 6B and 6C). We validated the defect in *Pdk4* activation in *Fgf21*^cLKO^ liver from fasting mice in both males and females (Figures 6D and S6B). KEGG pathway analysis also revealed similar fasting-induced transcriptomic pathway changes in *Fgf21*^cLKO^ and *Per1*^iLKO^ mice in comparison to those in control mice, including upregulation of cytochrome pathways (Figure S6C). Moreover, fasting *Fgf21*^cLKO^ mice exhibited normal serum glucose and liver fatty acid accumulation during fasting, as well as impaired serum fatty acid, ketone body, ORO staining, and intrahepatic TG accumulation when compared with fasting *Fgf21*^fl/fl^ mice (Figures S6D-S6G).

We finally quantified the extent to which exogenously administered FGF21 reconstitutes substrate selection observed in fasting *Per1*^iLKO^ mice. We treated *Per1*^fl/fl^ or *Per1*^iLKO^ mice with or without recombinant FGF21 protein after a 14 h + 2 h fast/refeed and subjected them to heavy-isotope metabolic tracing *in vivo* (Figure 6E). Although we observed no significant defects found in total hepatic metabolites involved in glycolysis and PDH-mediated pathway in *Per1*^iLKO^ mice during refeeding (Figures S6H and S6I), [^13^C_6_]glucose tracing revealed increased labeling in the glycolytic and PDH-mediated TCA cycle in fasting *Per1*^iLKO^ mice (Figures 6F and 6G). Moreover, treatment with recombinant FGF21 significantly reduced glycolytic and PDH-mediated TCA-cycle flux in *Per1*^iLKO^ mice but did not drive significant changes in fasting *Per1*^fl/fl^ mice (Figures 6F and 6G).

At the physiological level, we examined whether recombinant FGF21 administration would complement defective substrate selection in fasting *Per1*^cLKO^ mice. We treated mice at ZT21 (9 h post fasting), i.e., at the point of RER divergence observed previously when comparing *Per1*^fl/fl^ and *Per1*^cLKO^ fasted mice (Figure 5B). We quantified changes in substrate selection by RER throughout 5 h after injection (Figure 6H). Indeed, FGF21 complementation suppressed fasting RER and reconstituted *Pdk4* expression during fasting in *Per1*^cLKO^ mice (Figures 6H and 6I). These data together validate a *Per1-Fgf21* axis that mediates *Pdk4* induction and fuel selection during fasting (Figure 6J).

## DISCUSSION

Optimal substrate selection is critical for the growth, adaptation, and long-term survival of a species. A well-designed system accounts for acute substrate flux and yet is also temporally primed to anticipate the organism’s feeding/fasting and nocturnal/diurnal behaviors. We showed here that *Per1* is a candidate to execute both functions. *Per1* is a canonical clock gene,^45^ yet we show that *Per1* is both rhythmically expressed and induced by fasting, and each occurs independently of *Bmal1* and several other clock genes. However, *Per1* function extends beyond substrate selection, as we observed that *Per1* also drives autophagic flux and peripheral lipolysis in response to fasting. Finally, we elucidate an oxidative control mechanism in observing that hepatocyte *Fgf21* links *Per1* upregulation to transcriptional *Pdk4* activation and PDH phosphorylation to modulate TCA-cycle flux. This is supported by *in vivo* complementation data using both indirect calorimetry and *in vivo* substrate labeling after reconstituting FGF21 and blocking PDH. This pathway overall couples the carbohydrate-deficient hepatocyte to whole-organism shunting away from glucose metabolism.

Data herein indicate that *Per1*’s control over substrate selection, intriguingly, occurs autonomously and independently of *Bmal1* and other core clock genes. Equally importantly, however, circadian and metabolic inputs into *Per1* expression are approximately additive (Figure 4H). To that end, we first show that genetic knockdown of other clock genes in multiple *in vitro* and *in vivo* model systems—in *Per2-, Cry1-, Bmal1-,* and *Clock*-deficient hepatocytes and in *Bmal1*^LKO^ mice—fails to alter fasting-induced *Per1* expression. Second, *Bmal1*^LKO^ mice are phenotypically normal during fasting, despite the traditional view that *Bmal1* mediates *Per1* transcription within the circadian context. Our data indicate that suprachiasmatic nucleus control may not fully extend to peripheral clocks. Together, our data suggest that hepatocyte *Per1* is regulated by circadian input and yet exerts its metabolic function independent of these circadian inputs. This coupling of distinct input response within the same sensing factor (*Per1*) permits rapid, dynamic, yet finely tuned substrate control that integrates, yet separately accounts for, an organism’s circadian and metabolic states. Moreover, because the metabolic *Per1* response amplitude is greater than its circadian response amplitude (Figure 4H), we postulate that the metabolic state is the dominant *Per1* input. This is supported by data showing that exogenous treatment with recombinant FGF21 protein or PDH inhibitor CPI-613 can normalize the transcriptional and physiological phenotype of *Per1*^LKO^ mice. Nevertheless, we acknowledge that further work is required to further this *Per1-Fgf21-Pdk4* axis. This would include subsequent determination as to whether this signaling pathway invokes hepatocyte-intrinsic versus hepatocyte-extrinsic processes—or both—in executing its full physiological sequelae.

We also show that excluding carbohydrate from the hepatocyte is sufficient to induce *Per1*. Thus, the carbohydrate-specific sensing aspect of this pathway reveals important translational applications. We identified trehalose analogs 4-TA, 6-TreAz, and IMCTA, each comprising distinct carbon structures and each of which differentially and selectively induces *Per1* but not *Per2.* Our data support prior data indicating a structure-activity relationship linking trehalose-like compounds to induction of circadian and fasting-induced genes.^46^

In sum, we identify a fundamental control mechanism that integrates temporal and metabolic inputs to dictate whole-organism substrate selection and overall fasting adaptations through *Per1.* Because intracellular carbohydrate is a key signal to suppress this pathway, we now introduce pharmacological tools that can be used to examine normal hepatocyte circadian interactions with metabolic control.

### Limitations of the study

Some potential limitations to the study exist and should be high-lighted. First, our *in vitro* experiments that suggest *Bmal1* is dispensable for *Per1* upregulation and the downstream *Fgf21-Pdk4* axis may be in part confounded by the asynchronous state of the cells during the experiment. It should be noted, however, that data from these cultures were corroborated by the observation in our *in vivo Bmal1*^cLKO^ fasting time-course experiment, which also more directly demonstrated that *Bmal1* is not fully required for upregulation of this axis during fasting. Second, although we demonstrate some similarities in metabolic defects that are shared between hepatocyte-specific *Per1-* and *Fgf21-*deficient mice during fasting, we cannot conclude that these two models precisely phenocopy fasting metabolism, particularly with regard to the effects of *Per1* and *Fgf21* on mitochondrial function. This opens the possibility of a distinct fasting metabolic control profile mediated by *Per1*, which offers the opportunity for future interrogation.

## RESOURCE AVAILABILITY

### Lead contact

Further information and requests for resources and reagents should be directed to and will be fulfilled by Brian J. DeBosch (bdebosch@iu.edu).

### Materials availability

All unique/stable reagents generated in this study are available from the lead contact without restriction.

### Data and code availability

- Bulk RNA-seq and single-nucleus multiome sequencing data have been deposited in the NCBI Sequence Read Archive with accession number NCBI: PRJNA1161134.
- *In vivo* tracing metabolomic data are available from https://doi.org/10.21228/M8GR8V.
- This paper does not report original code.
- Any additional information required to reanalyze the data reported in this paper is available from the lead contact upon request.

## STAR ★ METHODS

### EXPERIMENTAL MODEL AND STUDY PARTICIPANT DETAILS

#### Mice

Wild-type C57BL/6J-strain mice (Jackson Laboratory, 000664), *Fgf121*^fl/fl^ mice (Jackson Laboratory, 022361) and *Bmal1*^fl/fl^ mice (Jackson Laboratory, 007668) were obtained directly from the Jackson Laboratory. *Per1*^fl/fl^ mice were generated by Genome Engineering & iPSC Center (GEiC) at Washington University. Upon arrival, mice were equilibrated for a minimum of 7 days in the specific pathogen-free vivarium prior to initiating metabolic measurements. Mice were kept under a 12 h alternating light/dark, temperature-controlled facility throughout the experimentation. All *in vivo* experimental procedures were performed in strict accordance with Institutional Animal Care and Use Committee (IACUC) guidelines at Washington University School of Medicine. Male mice were used unless specified in the figure legends. All experiments were done when mice are 8-week-old.

#### AML12 cell line

AML12 cells (CRL-2254) were purchased directly from the American Type Culture Collection (ATCC) and propagated and maintained precisely per manufacturer specification. For *in vitro* feed/starve experiment, 1*10^6^ cells (per well) were seeded in 6-well plates. After overnight attachment, cells were treated with 10^8^ plaque-forming units (PFU) of Ad-GFP or Ad-shPer1 purchased directly from Vector Biolabs. 48 h post-transduction, culture media was switched to either complete media (Dulbecco’s modified Eagle’s medium/nutrient mixture F-12 (DMEM/F12; ThermoFisher, 11,320–033) supplemented with 10% fetal bovine serum (Gibco, 26140079), 40 ng/mL dexamethasone (Sigma Aldrich, D4902) and insulin-transferrin-selenium solution (Sigma, I1884)) or starvation media (DMEM low glucose, pyruvate; Gibco, 11885084 supplemented with 40 ng/mL dexamethasone). For autophagic flux quantification in Figure 1I, media was supplemented with DMSO as vehicle control or 200 nM bafilomycin for 6 h. For *in vitro* experiment in Figure 5, cells were transduced with Ad-GFP or Ad-shPer1 for 48 h and followed by 48 h media switch to complete or starvation media. Seahorse XF Cell Mito Stress (Agilent, 103015-100), XF Long Chain Fatty Acid Oxidation Stress (Agilent, 103672-100) and XF Glucose/ Pyruvate Oxidation Stress (Agilent, 103673-100) tests were performed according to manufacturer’s directions.

For *in vitro* experiment knocking down *Fgf21* or other core circadian genes, cells were transfected with ASO purchased from INOS Pharmaceuticals (*Fgf21* ASO, INO-256617), or siRNA purchased directly from Santa Cruz Biotechnology (si*Bmal1*, sc-38166; si-Clock, sc-35075; and siCry1, sc-44835). Transfection was performed according to the RNAiMAX (Invitrogen, 13778150) transfection protocol. 72 h post-transfection, culture media was switched to either complete media or starvation media for 6 h. For *in vitro* treatment of CPI-613 in AML 12 cells, 25μM CPI-613 (Sigma-Aldrich, SML0404) was added 2 h prior to harvest.

#### Primary hepatocyte isolation, culture and treatment

Primary murine hepatocytes obtained from wildtype, *Per2*^KO[20]^ mice were isolated and maintained in regular DMEM growth media (Sigma, D5796) containing 10% FBS, as previously reported.^47,48^ For *in vitro* starvation experiment in isolated primary hepatocytes, DMEM low glucose, pyruvate (Gibco, 11885084) was used.

### METHOD DETAILS

#### Virus injection

Adeno-associated viruses under TBG promoter overexpressing GFP (AAV8-TBG-GFP) and Cre (AAV8-TBG-Cre) were obtained as ready-to-use viral stock from Vector Biolabs (Malvern, PA, USA). 10^11^ viral particles were injected via tail vein 10–14 days prior to fast/refeed treatment. Standard rodent chow diet was used throughout the study.

#### Serum and hepatic lipids, indirect calorimetry

For all serum analyses, submandibular blood was collected immediately prior to sacrifice and serum was separated. Triglycerides (ThermoFisher, TR22421), free fatty acids (Wako Diagnostics, 999–34691, 995–34791, 991–34891, 993–35191), glucose (Cayman, 10009582) and ketone body (Cayman, 700190) quantification were performed using commercially available reagents according to manufacturer’s directions. Hepatic lipids, histology and indirect calorimetry analyses are done as previously reported.^30^ Glucose oxidation was calculated using formula ((4.585*VCO_2_)–(3.226*VO_2_))*4.^49^ For *in vivo* injections of CPI-613 (Sigma-Aldrich, SML0404) and FGF21 (Bio-techne, 8409-FG/CF-MTO), mice were intraperitoneally injected 25 mg/kg and 1 mg/kg body weight correspondingly.

#### Quantitative real-time PCR (qRT-PCR)

Total RNA was prepared by homogenizing snap-frozen livers or cultured cells in Trizol reagent (Invitrogen, 15596026) according to the manufacturer’s protocol. cDNA was prepared using Qiagen Quantitect reverse transcriptase kit (Qiagen, 205310). Real-time qRT-PCR was performed with Step-One Plus Real-Time PCR System (Applied Biosystems) using SYBR Green master Mix Reagent (Applied Biosystems) and specific primer pairs. Relative gene expression was calculated by a comparative method using values normalized to the expression of the internal control gene *β-Actin.* All primers were custom-synthesized oligonucleotides obtained from Integrated DNA Technologies. Primer sequences are included in Table S3.

#### Immunoblotting

Protein from tissues and cells were harvested and proceeded as previously reported.^30^ Antibodies information is provided in key resources table.

#### *In vivo* tracing and metabolomic analysis

All mouse studies were approved by the Institutional Care and Use Committee at Washington University in Saint Louis. To perform infusion studies, a catheter (Instech, C20PU-MJV1301) was placed in the right jugular vein and connected to a vascular access button (Instech, VABM1B/25) implanted subcutaneously in the back of the mice. All surgeries were performed at the Hope Center for Neurological Diseases, Washington University. Mice were allowed to recover from surgery for at least one week before tracer infusion.

U^13^C-Glucose (CIL, CLM-1396-PK) was freshly prepared in saline at a concentration of 200mM. The mice were weighed to calculate the tracer infusion rate. To begin infusion, the vascular access button of individual mice was connected to the infusion line with a swivel (Instech, SMCLA), tether (Instech, KVABM1T/25), and infusion pump (CHEMYX, Fusion 100T). The infusion line was prefilled with 200mM U^13^C-Glucose. Prime infusion was initiated at 1 μL/min/g for 2 min to clear the catheter locking solution, followed by continued infusion at 0.1 μL/min/g for 2 h. Following completion of the glucose infusion, mice were anesthetized, and blood was collected by cardiac puncture. Tissues were subsequently collected as quickly as possible (in 10 min or less) following euthanasia and snap-frozen in liquid nitrogen. Tissues were stored at −80°C until processing for LC/MS analysis.

The liver tissue was mixed with ice-cold methanol:acetonitrile:water (2:2:1), and subjected to two cycles of 7 m/s (30 s/cycle) using an Omni Bead Ruptor Elute Homogenizer. For every 1 mg of tissue wet weight, 40 μL of extraction solvent was added. Samples were then incubated at −20°C for 1 h to precipitate protein. Tissue extracts were centrifuged at 20,000 g and 4°C for 10 min, and the supernatant was transferred into LC/MS vials.

Ultra-high-performance LC (UHPLC)/MS was performed with a Thermo Scientific Vanquish Horizon UHPLC system interfaced with a Thermo Scientific QExactive Plus Mass Spectrometer. Polar metabolites were separated on a HILICON iHILIC-(P)-Classic column (100 × 2.1 mm, 5 μm). The mobile-phase solvents were composed of: A = 20 mM ammonium bicarbonate, 2.5 μM medronic acid, 0.1% ammonium hydroxide in water:acetonitrile 95:5; and B = water:acetonitrile 5:95. The column compartment was maintained at 40°C. The following linear gradient was applied at a flow rate of 0.25 mL min^−1^: 0 – 1min, 90% B; 12min, 35% B; 12.5–14.5min, 25% B; 15min, 90% B followed by a re-equilibration phase of 10 column volumes. The injection volume was 4 μL for all polar experiments. Data was acquired in positive and negative ion mode with the following settings: spray voltage, 3.5 kV (positive) and −2.8 kV (negative); sheath gas, 45; auxiliary gas, 10; sweep gas, 2; capillary temperature, 250°C; aux gas temperature, 300°C; mass range, 65–975 Da; resolution, 140,000. LC/MS data were processed and analyzed with the open-source Skyline software.^50^ Natural-abundance correction of ^13^C was performed with AccuCor.^51^ Data can be found in Metabolomics Workbench Project PR002144.^52^

#### RNA-seq and snMultiome-seq

RNA sequencing was performed by the Washington University Genome Technology Access Center (GTAC) as we reported.^30^ Differentially expressed genes from the heatmap was analyzed by edgeR package,^53-55^ and the heatmap was generated by pheatmap package.^56^ Liver nuclei for snMultiome-seq were isolated using chromium nuclei isolation kit with RNase inhibitor (10x genomics, 1000494). Isolated nuclei were processed and sequenced by Washington University GTAC, and data were analyzed by Seurat and Signac package.^57,58^

### QUANTIFICATION AND STATISTICAL ANALYSIS

#### Statistical analysis

Data are presented as the mean ± SEM. The number of independent biological samples (n) in each experiment is detailed in the figure legends. The data were analyzed by unpaired two-sided Student’s t test, one-way ANOVA or two-way ANOVA with GraphPad Prism 9 software as specified in the figure legends. For all the analyses, */a/#*p* < 0.05, **/aa/##*p* < 0.01, ***/aaa/###*p* < 0.001, ****/aaaa/####*p* < 0.0001.

## Supplementary Material

1

## Figures and Tables

**Table:** KEY RESOURCES TABLE

REAGENT or RESOURCE	SOURCE	IDENTIFIER
Antibodies		
β-ACTIN	Cell Signaling Technology	Cat#3700 RRID:AB_2242334
FGF21	abcam	Cat#171941 RRID:AB_2629460
GFP	Cell Signaling Technology	Cat#2956 RRID:AB_1196615
LC3A/B	Cell Signaling Technology	Cat#12741 RRID:AB_2617131
PDH	Cell Signaling Technology	Cat#2784 RRID:AB_2162928
PDK4	proteintech	Cat#12949-1-AP RRID:AB_2161499
Pyruvate Dehydrogenase	Cell Signaling Technology	Cat#2784 RRID:AB_2162928
phospho-PDHα1(Ser293)	Cell Signaling Technology	Cat#31866 RRID:AB_2799014
phospho-ULK1(Ser317)	Cell Signaling Technology	Cat#37762 RRID:AB_2922992
phospho-ULK1(Ser757)	Cell Signaling Technology	Cat#14202 RRID:AB_2665508
ULK1	Cell Signaling Technology	Cat#8054 RRID:AB_11178668
VINCULIN	Cell Signaling Technology	Cat#13901 RRID:AB_2728768
Anti-mouse IgG, HRP-linked	Cell Signaling Technology	Cat#7076; RRID:AB_330924
Anti-rabbit IgG, HRP-linked	Cell Signaling Technology	Cat#7074; RRID:AB_2099233
Bacterial and virus strains		
AAV8-TBG-GFP	Vector Biolabs	SKU#Vb1743
AAV8-TBG-Cre	Vector Biolabs	SKU#VB1724
Ad-GFP	Vector Biolabs	Cat#1060
Ad-shPer1	Vector Biolabs	SKU#shADV-268400
Chemicals, peptides, and recombinant proteins		
U^13^C-Glucose	Cambridge Isotope Laboratories	Cat#CLM-1396-10
FGF21 recombinant protein	Bio-techne	Cat#8409-FG
6,8-Bis(benzylthio)-octanoic acid (CPI-613)	Sigma-Arch	Cat#SML0404
Critical commercial assays		
Mouse/Rat Fibroblast Growth Factor 21 ELISA	BioVendor R&D	Cat#RD291108200R
Infinity^™^ Triglycerides	Thermo	Cat#TR22421
HR Series NEFA-HR	FUJIFILM	Cat#999–34691, 995–34791, 991–34891, 993-35191
Glucose Colorimetric Assay Kit	Cayman	Cat#10009582
β-Hydroxybutyrate (Ketone Body) Colorimetric Assay Kit	Cayman	Cat#700190
Seahorse XF Cell Mito Stress Kit	Agilent Technologies	Cat#103015-100
Seahorse XF Long Chain Fatty Acid Oxidation Stress Kit	Agilent Technologies	Cat#103672-100
Seahorse XF Glucose/Pyruvate Oxidation Stress Kit	Agilent Technologies	Cat#103673-100
Experimental models: Cell lines		
AML12	ATCC	Cat#CRL-2254
Deposited data		
*In vivo* tracing metabolomic result	This paper	Metabolomics Workbench: PR002144
RNA-sequencing and single-nuclei multiome sequencing	This paper	SRA: PRJNA1161134
Experimental models: Organisms/strains		
*Mouse*: *Per1*^fl/fl^	This paper	This paper
*Mouse*: *Bmal1*^fl/fl^	Jackson Laboratory	Strain#007668 RRID:IMSR_JAX:007668
*Mouse*: *Per2*^KO^	Bae et al., 2001^20^	From Dr. E.D. Herzog
*Mouse*: *Fgf21*^fl/fl^	Jackson Laboratory	Strain#022361 RRID:IMSR_JAX:022361
*Mouse*: C57BL/6J	Jackson Laboratory	Strain#000664 RRID:IMSR_JAX:000664
*Mouse*: Alb-Cre	Jackson Laboratory	Strain#003574 RRID:IMSR_JAX:003574
Oligonucleotides		
si*Bmal1*	Santa Cruz Biotechnology	Cat#sc-38166
siClock	Santa Cruz Biotechnology	Cat#sc-35075
siCry1	Santa Cruz Biotechnology	Cat#sc-44835
si*Ppara*	Santa Cruz Biotechnology	Cat#sc-36380
si*Esrrg*	Santa Cruz Biotechnology	Cat#sc-44705
si*Atf1*	Santa Cruz Biotechnology	Cat#sc-29755
*Fgf21* antisense oligonucleotide (ASO)	IONIS Pharmaceuticals	Cat#ION-256,617
qRT-PCR sequences	Table S3	N/A
Software and algorithms		
ImageJ software	https://imagej.nih.gov/ij.	https://imagej.nih.gov/ij.
Image lab software	BIO-Rad Laboratories	N/A
GraphPad Prism 9 software	GraphPad	N/A
Graphic abstract and schematics in Figures 4 and 6 were created using biorender.com	https://www.biorender.com/	N/A

## Highlights

- Hepatocyte *Per1* is induced by fasting independent of the core circadian clock
- Hepatocyte *Per1*-deficient mice exhibit broadly defective fasting physiology
- *Per1* mediates fasting-induced *Fgf21* and *Pdk4* expression
- FGF21 reconstitution and PDH inhibition rescue defective fasting metabolism
