# Supplementary material for: Hepatocyte Period 1 dictates oxidative substrate selection independent of the core circadian clock
Source: Cell Rep. Author manuscript; Available in PMC 2024 Nov 27. (PMC11601098; doi:10.1016/j.celrep.2024.114865)
Supplement: 1 [file NIHMS2031324-supplement-1.pdf]

Cell Reports, Volume *43*

Supplemental information

Hepatocyte *Period 1* dictates oxidative substrate selection independent of the core circadian clock

Jiameng Sun, Yiming Zhang, Joshua A. Adams, Cassandra B. Higgins, Shannon C. Kelly, Hao Zhang, Kevin Y. Cho, Ulysses G. Johnson, Benjamin M. Swarts, Shun-Ichi Wada, Gary J. Patti, Leah P. Shriver, Brian N. Finck, Erik D. Herzog, and Brian J. DeBosch

Supplemental Information

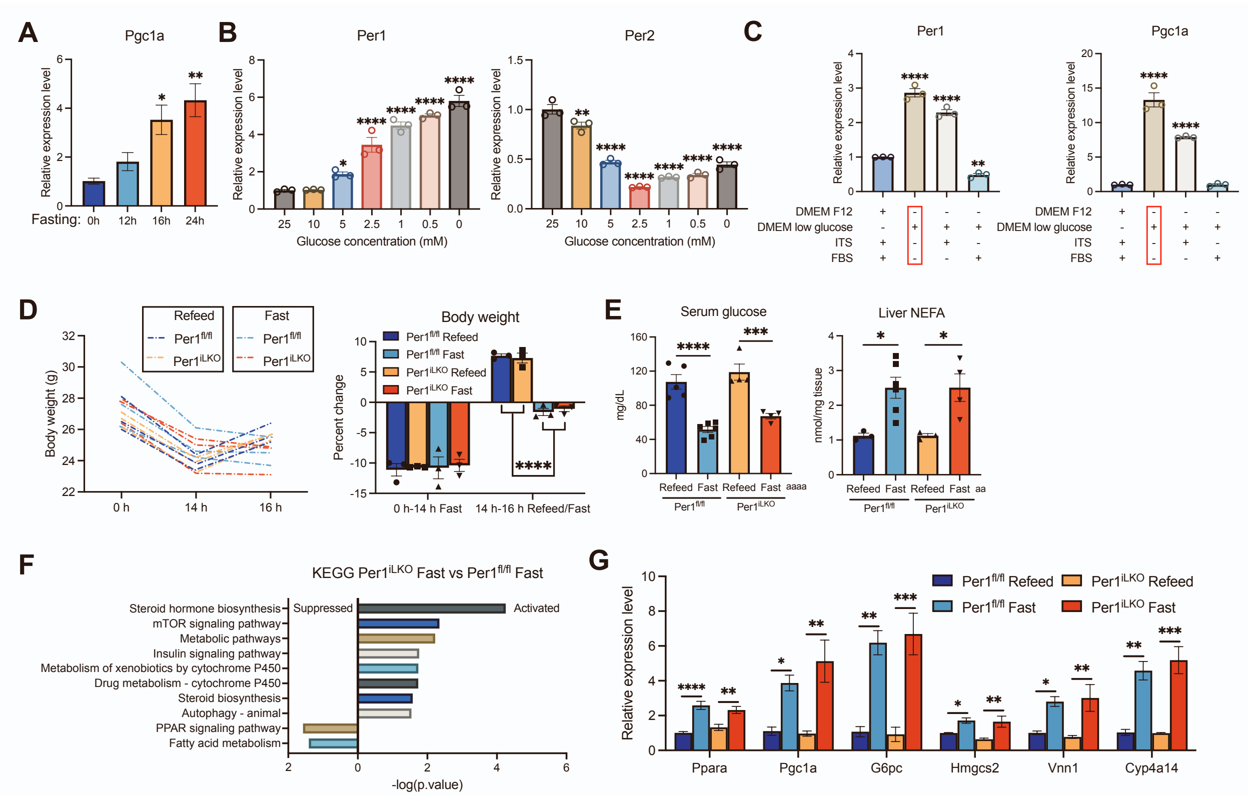

Figure S1. Hepatocyte *Per1* deficiency enhances thermogenesis and gas exchange during fasting.

Related to Figure 1

(A) Relative expression level of *Pgc1a* in mice fasted for 0, 12, 16 or 24 h; n=3.

(B) Relative expression level of *Per1* and *Per2* in culture media with different glucose concentration; n=3.

(C) Relative expression level of *Per1* and *Pgc1a* in culture media with different components; n=3.

(D) Individual total body weight change during 14 h+2 h fast/refeed in $Per1^{fl/fl}$ and $Per1^{iLKO}$ mice (left), and percent body weight change during 14 h fasting and 2 h refeeding/fasting (right); n=3.

(E) Serum glucose (left) and hepatic NEFA level (right) from mice in Fig. 1K; n=3-6.

(F) KEGG pathway analysis on differentially expressed gene between fasted liver from $Per1^{iLKO}$ and $Per1^{fl/fl}$ mice in Fig. 1K; n=3.

(G) Relative expression level of *Ppara* and its downstream targets from mice in Fig. 1K; n=3.

Data expressed as mean ± SEM. */a/# p<0.05, **/aa/## p<0.01, ***/aaa/### p<0.001, ****/aaaa/#### p<0.0001 by student's t-test (A, C). one-way ANOVA (B) and two-way ANOVA (D,E,G)

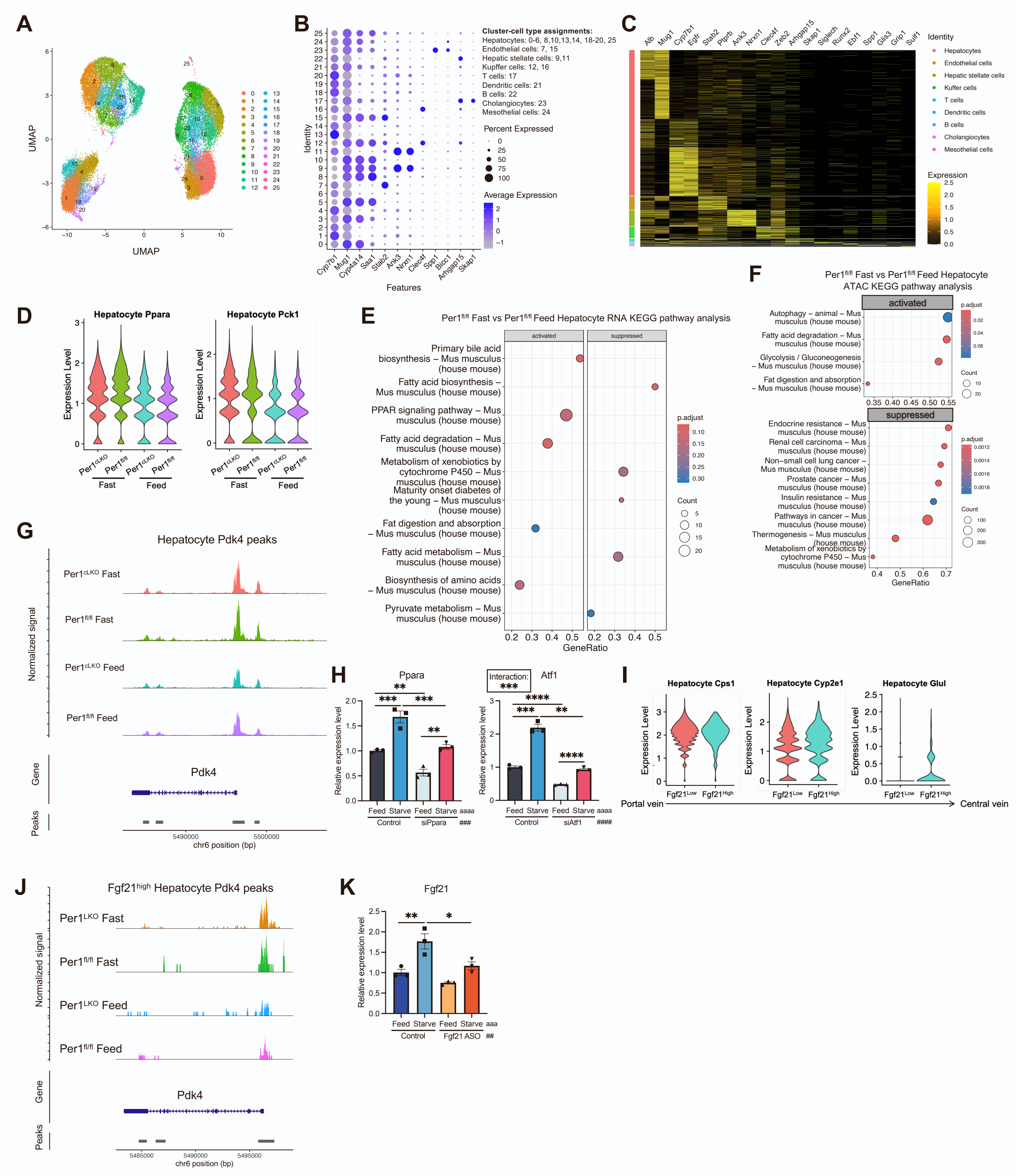

Figure S2. Single-multiome sequencing reveals distinct transcriptomic profile in selected hepatocyte subpopulation between *Per1*^{cLKO} and *Per1*$^{fl/fl}$ mice. Related to Figure 2 and 3.

(A) 26 clusters identified in nuclei isolated from *Per1*$^{fl/fl}$ and *Per1*^{cLKO} (*Per1*$^{fl/fl}$, Alb-Cre) liver either *ad lib* fed or 16 h-fasted; n=3.

(B) Genetic markers used for assigning major hepatic cell types in each cluster.

(C) Heatmap of cell markers in each assigned hepatic cell type.

(D) Relative expression level of *Ppara* and *Pck1* in hepatocyte population from each group.

(E) KEGG pathway analysis on differentially expressed genes between *Per1*$^{fl/fl}$ fasted and *Per1*$^{fl/fl}$ *ad lib* fed hepatocytes.

(F) KEGG pathway analysis on differentially expressed peak signals between *Per1*$^{fl/fl}$ fasted and *Per1*$^{fl/fl}$ *ad lib* fed hepatocytes.

(G) Coverage plot at *Pdk4* locus from hepatocyte population.

(H) Relative expression level of *Ppara* and *Atf1* in AML12 cells treated with siPpara or siAtf1 in Fig. 2H; n=3.

(I) Relative expression level of liver zonality markers *Cps1*, *Cyp2e1* and *Glul* in defined *Fgf21*^{High} and *Fgf21*^{Low} population.

(J) Coverage plot at *Pdk4* locus from selected *Fgf21*^{High} hepatocyte subpopulation.

(K) Relative expression level of *Fgf21* in AML12 cells treated with Fgf21 ASO in Fig. 3H-I; n=3.

Data expressed as mean ± SEM. */a/# p<0.05, **/aa/## p<0.01, ***/aaa/### p<0.001, ****/aaaa/#### p<0.0001 by two-way ANOVA (H and K).

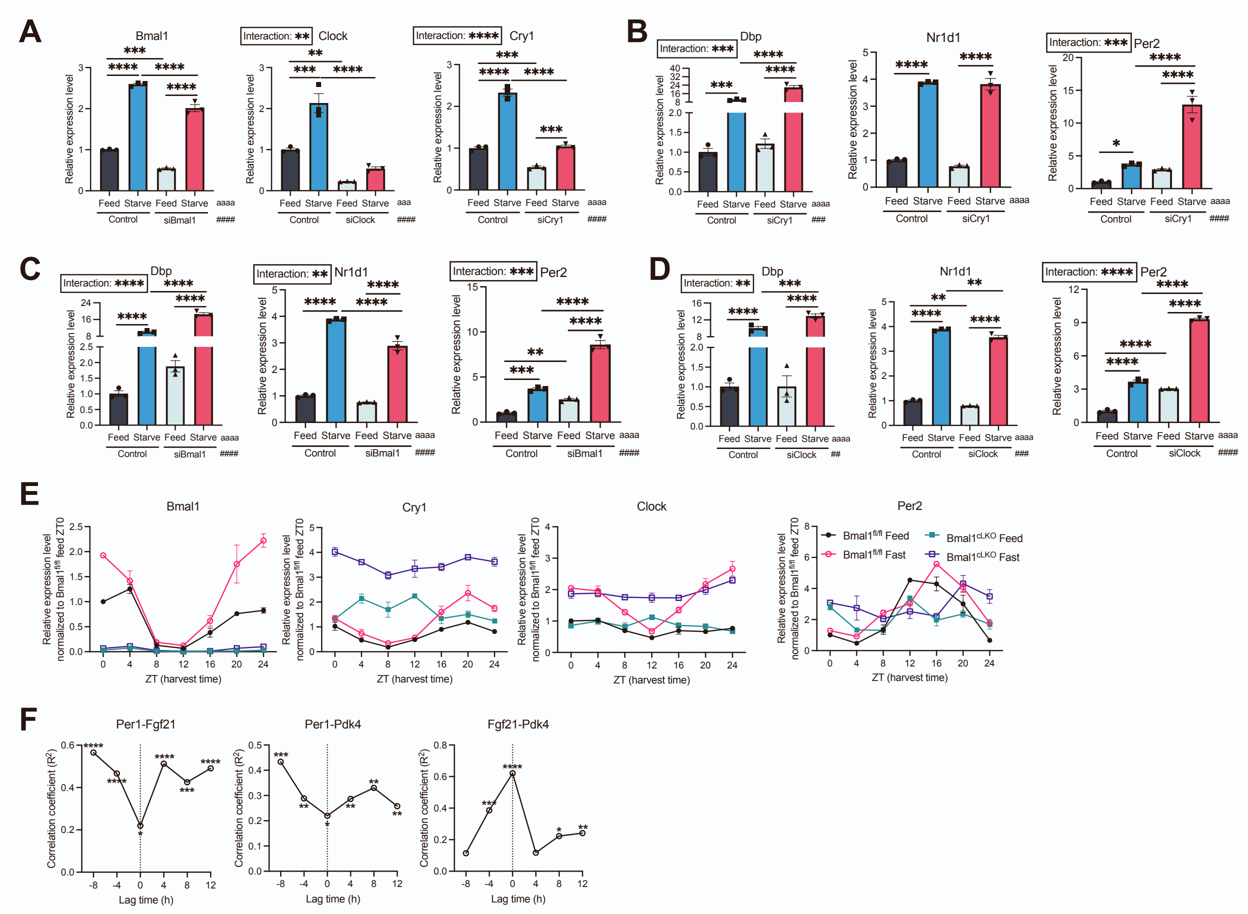

Figure S3. Liver *Per1-Fgf21-Pdk4* expression levels are strongly correlated. Related to Figure 4.

(A) Relative expression level of *Bmal1*, *Clock* and *Cry1* from AML12 cells treated with siRNA targeting *Bmal1*, *Clock* or *Cry1*; n=3.

(B-D) Relative expression level of *Dbp*, *Nr1d1* and *Per1* in AML12 cells from Fig. 4A.

(E) Relative expression level of *Bmal1*, *Cry1*, *Clock* and *Per2* (normalized to ZT0 *Bmal1*$^{fl/fl}$ feed) from mice during 16 h-fasting timecourse in Fig. 4G; n=3-4.

(F) Correlation test result at different lag times (i.e. negative lag time represents a shift of expression towards left, and vice versa) between *Per1* and *Fgf21* (left), *Per1* and *Pdk4* (middle) and *Fgf21* and *Pdk4* (right) expression when normalized to ZT0 *Bmal1*$^{fl/fl}$ feed.

Data expressed as mean ± SEM. */a/# p<0.05, **/aa/## p<0.01, ***/aaa/### p<0.001, ****/aaaa/#### p<0.0001 by two-way ANOVA (A-D) and Pearson correlation test (F).

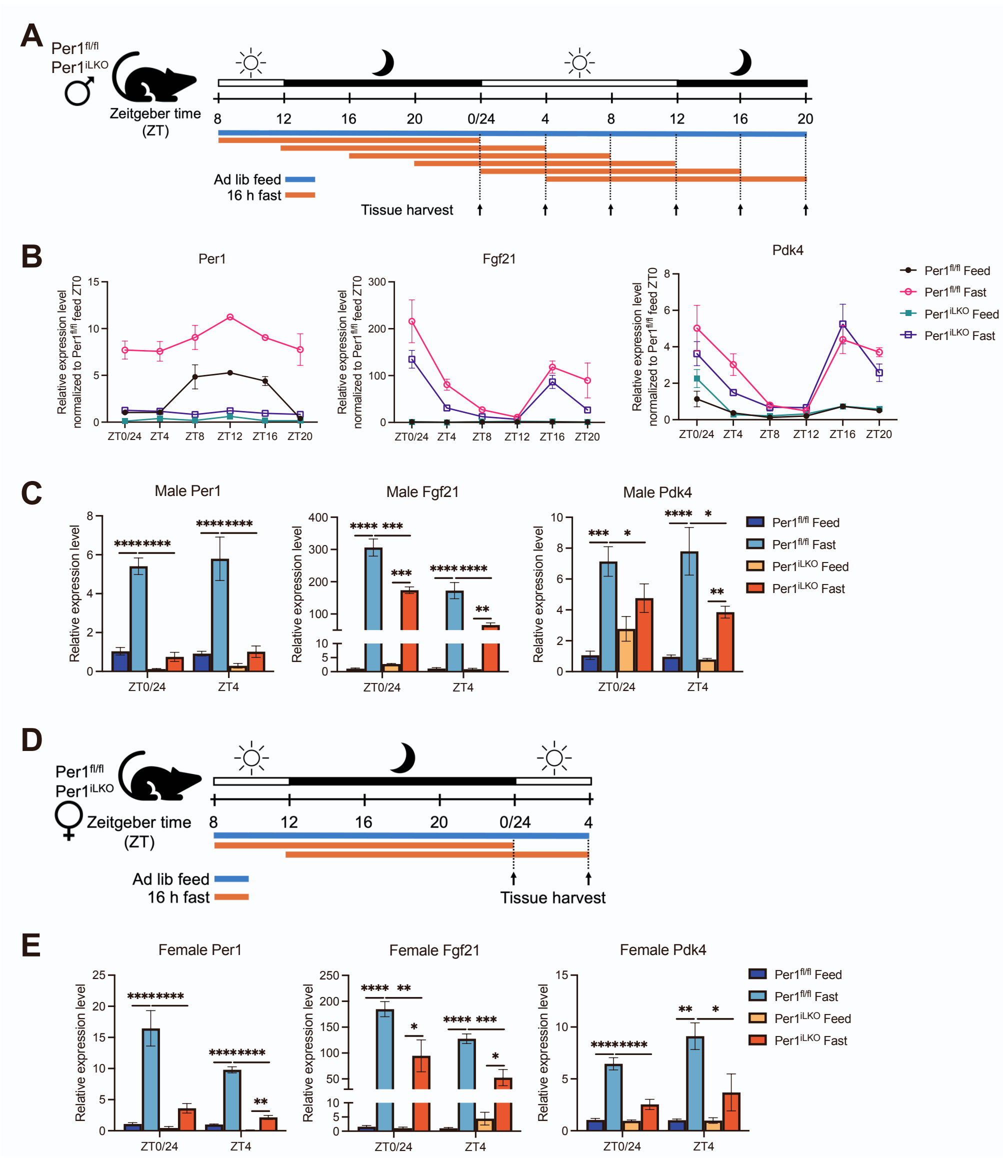

Figure S4. *Per1*^{iLKO} dark-phase fasted mice show impaired *Fgf21* and *Pdk4* in both male and female. Related to Figure 4.

(A) Schematic of *in vivo* timecourse experiment in *Per1*$^{fl/fl}$ and *Per1*^{iLKO} male mice. Mice were either *ad lib* fed or fasted for 16 h, and liver tissues were harvested every 4 h in a 24 h duration; n=3-5.

(B) Relative expression level of *Per1*, *Fgf21* and *Pdk4* in male mice fed/fasted; n=3-5.

(C) Relative expression level of *Per1*, *Fgf21* and *Pdk4* in male mice fed/fasted during dark phase; n=3-5.

(D) Schematic of *in vivo* timecourse experiment in *Per1*$^{fl/fl}$ and *Per1*^{iLKO} female mice that were fasted/fed during dark phase.

(E) Relative expression level of *Per1*, *Fgf21* and *Pdk4* in female mice fed/fasted; n=3-6.

Data expressed as mean ± SEM. */a/# p<0.05, **/aa/## p<0.01, ***/aaa/### p<0.001, ****/aaaa/#### p<0.0001 by two-way ANOVA (C, E).

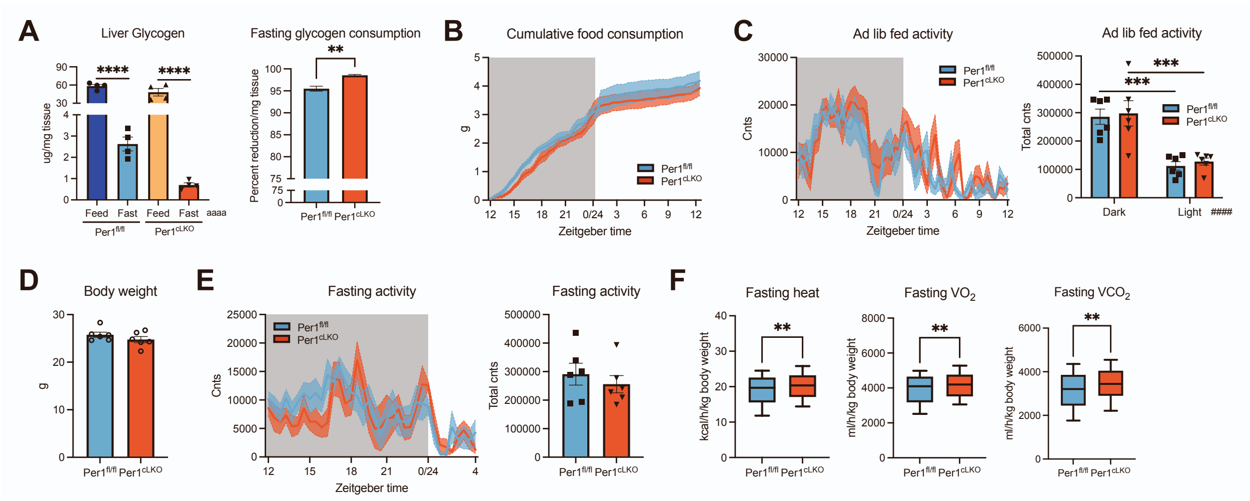

Figure S5. Hepatoycte *Per1* mediates energy metabolism during fasting. Related to Figure 5.

(A) Hepatic glycogen level in *Per1*$^{fl/fl}$ and *Per1*^{cLKO} (Alb-Cre) mice 16h-fasted/ad lib fed (left). Percent hepatic glycogen level change from 16 h-fasted mice compared to *ad lib* fed mice (right); n=3.

(B) Cumulative food consumption in *Per1*$^{fl/fl}$ and *Per1*^{cLKO} mice; n=6.

(C) Activity measured by beam counts during 24 h *ad lib* feeding in *Per1*$^{fl/fl}$ and *Per1*^{cLKO} mice (left), and total activity quantification during 12 h dark and 12 h light phase (right); n=6.

(D) Total body weight of *Per1*$^{fl/fl}$ and *Per1*^{cLKO} mice prior to fasting; n=6.

(E) Activity measured by beam counts during 16 h fasting in *Per1*$^{fl/fl}$ and *Per1*^{cLKO} mice (left), and total activity quantification (right); n=6.

(H) Fasting heat, O_2 consumption and CO_2 production; n=6.

Data expressed as mean ± SEM. */a/# p<0.05, **/aa/## p<0.01, ***/aaa/### p<0.001, ****/aaaa/#### p<0.0001 by two-way ANOVA (A left, C) and student's t-test (A right, F).

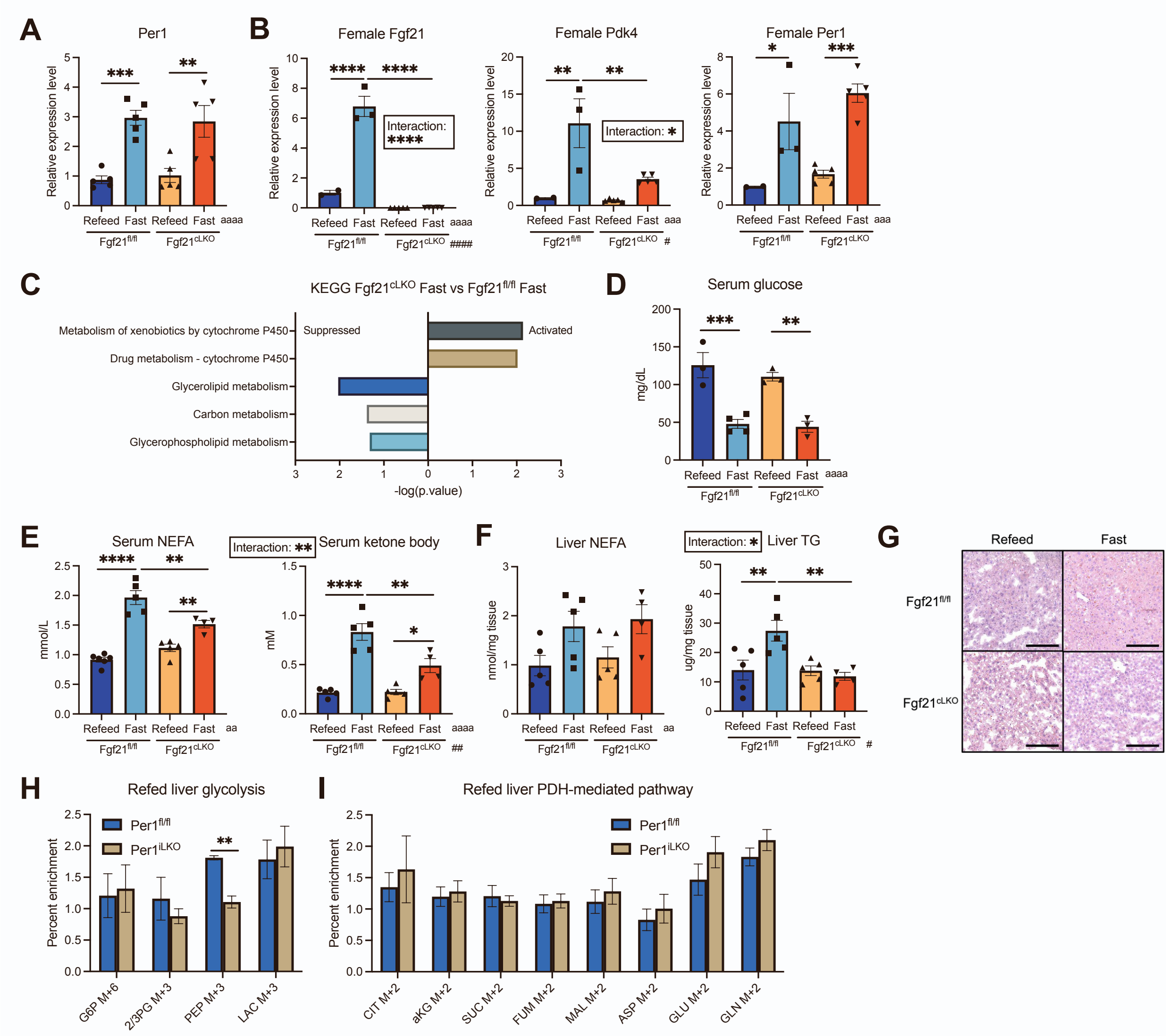

Figure S6. *Fgf21*^{LKO} fasted mice show similar defects as in *Per1*^{LKO} fasted mice. Related to Figure 6.

(A) Relative expression level of liver *Per1* from male *Fgf21*$^{fl/fl}$ or *Fgf21*^{cLKO} (*Fgf21*$^{fl/fl}$, Alb-Cre) mice underwent 14 h+2 h fast/refeed; n=5.

(B) Relative expression level of liver *Per1* from female *Fgf21*$^{fl/fl}$ or *Fgf21*^{cLKO} mice underwent 14 h+2 h fast/refeed; n=2-5.

(C) KEGG pathway analysis on differentially expressed genes between fasted liver from *Fgf21*^{cLKO} and *Fgf21*$^{fl/fl}$ mice in Fig. 6A; n=3.

(D) Serum glucose level in *Fgf21*$^{fl/fl}$ and *Fgf21*^{cLKO} mice fasted/refed; n=3.

(E) Serum NEFA (left) and ketone body (right) level in *Fgf21*$^{fl/fl}$ and *Fgf21*^{cLKO} mice fasted/refed; n=4-5.

(F) Hepatic NEFA (left) and TG (right) level in *Fgf21*$^{fl/fl}$ and *Fgf21*^{cLKO} mice fasted/refed; n=4-5.

(G) Representative ORO-staining in *Fgf21*$^{fl/fl}$ and *Fgf21*^{cLKO} mice fasted/refed.

(H) Percent enreichment in correpondingly labelled metabolites involved in glycolysis in *Per1*$^{fl/fl}$ and *Per1*^{iLKO} refeeding mice from Fig. 6E; n=3.

(I) Percent enreichment in correpondingly labelled metabolites involved in PDH-mediated TCA cycle in *Per1*$^{fl/fl}$ and *Per1*^{iLKO} refeeding mice; n=3.

Data expressed as mean ± SEM. */a/# p<0.05, **/aa/## p<0.01, ***/aaa/### p<0.001, ****/aaaa/#### p<0.0001 by two-way ANOVA (A, B, D-F) and one-way ANOVA (H).

Table S1. Enriched motifs in hepatocyte population. Related to Figure 2.

Group	gene	RNA.auc	RNA.pval	Motif.feature	Motif.auc	Motif.pval	Avg.auc
Per1^{cLKO} Fast	Esrrg	0.5262830	2.645990e-17	MA0643.1	0.5612446	7.005605e-48	0.5437638
	Atf1	0.5065614	2.728539e-03	MA0604.1	0.5437431	2.955275e-25	0.5251523
Per1^{cLKO} Feed	Klf12	0.6146984	8.689825e-171	MA0742.1	0.5564455	1.818254e-40	0.5855720
	Nr5a2	0.5948772	1.008387e-148	MA0505.1	0.5596291	5.873644e-45	0.5772532
	Sox6	0.6004311	5.546224e-154	MA0515.1	0.5139823	9.700927e-04	0.5572067
	Rxra	0.5663988	9.272025e-71	MA0512.2	0.5450215	2.339248e-26	0.5557102
	Tcf12	0.5402364	7.656487e-30	MA0521.1	0.5525068	3.007481e-35	0.5463716
	Rarb	0.5086031	4.386500e-06	MA0857.1	0.5693744	3.206857e-60	0.5389888
	Smad4	0.5198845	5.679083e-16	MA1153.1	0.5555648	2.877042e-39	0.5377246
	Esrrg	0.5442171	1.884936e-45	MA0643.1	0.5295935	2.900795e-12	0.5369053
	Arnt	0.5217373	5.620737e-15	MA0004.1	0.5509473	2.763823e-33	0.5363423
	Nr2f6	0.5237999	6.176229e-34	MA0677.1	0.5459375	2.256043e-27	0.5348687
	Zfx	0.5138882	7.766332e-11	MA0146.2	0.5489480	7.463823e-31	0.5314181
	Foxj3	0.5115395	1.710666e-08	MA0851.1	0.5488136	1.078898e-30	0.5301766
	Arid3b	0.5052334	7.402376e-05	MA0601.1	0.5477828	1.761168e-29	0.5265081
	Prdm15	0.5068935	6.873197e-05	MA1616.1	0.5431109	2.649330e-24	0.5250022
	Creb3l2	0.5200848	1.265883e-14	MA0608.1	0.5278237	5.207803e-11	0.5239542
	Sox5	0.5207360	5.845664e-07	MA0087.1	0.5211047	6.373013e-07	0.5209204
	Alx1	0.5004074	5.967546e-03	MA0854.1	0.5318171	6.045335e-14	0.5161123
	Dlx3	0.5003035	5.040270e-03	MA0880.1	0.5285888	1.526077e-11	0.5144462
	Dmrt1	0.5007911	2.044610e-03	MA1603.1	0.5276611	6.732404e-11	0.5142261
	Foxj2	0.5035124	2.191659e-03	MA0614.1	0.5223122	1.406037e-07	0.5129123
Per1$^{fl/fl}$ Feed	Foxj3	0.5076392	5.029668e-06	MA0851.1	0.5815316	2.742383e-122	0.5445854
	Sox6	0.5398223	1.378399e-37	MA0515.1	0.5429136	3.440396e-35	0.5413680
	Stat2	0.5122329	1.357782e-07	MA1623.1	0.5642389	1.205943e-76	0.5382359
	Npas2	0.5044391	5.534952e-11	MA0626.1	0.5198758	9.869345e-09	0.5121575
Per1$^{fl/fl}$ Fast	N/A						

Table S2. Differentially expressed genes in Fgf21^{High} versus Fgf21^{Low} hepatocytes. Related to Figure 3.

Per1$^{fl/fl}$ Feed Hepatocyte Fgf21^{High} vs Fgf21^{Low}			
	p_val	avg_log2FC	p_val_adj
Zfp985	4.70E-15	5.9853676	1.11E-10
Rasl2-9	4.70E-15	5.9853676	1.11E-10
Gm47257	4.70E-15	5.9853676	1.11E-10
Ctrc	3.61E-12	5.570330101	8.55E-08
Dipk1c	3.63E-12	5.248402006	8.60E-08
Cdc7	3.07E-10	5.248402006	7.28E-06
Ptprh	3.07E-10	5.248402006	7.28E-06
Gm48903	3.07E-10	5.248402006	7.28E-06
Gm36037	7.84E-10	3.9853676	1.86E-05
Ankrd35	7.43E-09	4.9853676	0.000176091
Gm3764	7.46E-09	4.762975179	0.000176783
A830082K12Rik	1.35E-08	2.962999787	0.000320366
Platr16	2.62E-08	4.078477005	0.0006216
Mlf1	8.17E-08	4.762975179	0.001935678

Galnt17	8.65E-08	3.284927882	0.002050508
Tnip2	4.93E-07	1.9853676	0.011681936
Hm629797	5.12E-07	3.897904759	0.012145709
Gpr143	5.30E-07	4.570330101	0.012567436
**Per1^{cLKO} Feed Hepatocyte Fgf21^{High} vs Fgf21^{Low}**			
Fgf6	1.23E-17	6.776830055	2.92E-13
Klrb1a	1.23E-17	6.776830055	2.92E-13
Gm44053	5.73E-12	5.776830055	1.36E-07
Gm6878	5.73E-12	5.776830055	1.36E-07
Gm35438.1	5.73E-12	5.776830055	1.36E-07
Gm15932	2.96E-10	4.191867554	7.01E-06
Dchs1	4.07E-09	5.191867554	9.64E-05
BC053393	4.07E-09	5.191867554	9.64E-05
Gm48898	4.07E-09	5.191867554	9.64E-05
Gm30655	4.07E-09	5.191867554	9.64E-05
Siah3	4.07E-09	5.191867554	9.64E-05
B130046B21Rik	4.07E-09	5.191867554	9.64E-05
Fblim1	1.07E-08	4.384512632	0.000254304
Eqtn	2.14E-07	4.776830055	0.005066062
Vmn2r60	2.14E-07	4.776830055	0.005066062
Il17d	2.14E-07	4.776830055	0.005066062
Mgat3	2.14E-07	4.776830055	0.005066062
Rmi2	2.14E-07	4.776830055	0.005066062
Tmem47	2.14E-07	4.776830055	0.005066062
Gm15046	2.14E-07	4.776830055	0.005066062
Gpc3	3.90E-07	4.021942553	0.009235809
Cd300c2	1.50E-06	3.869939459	0.035671042
**Per1$^{fl/fl}$ Fast Hepatocyte Fgf21^{High} vs Fgf21^{Low}**			
Alox12b	1.59E-14	6.498831079	3.78E-10
Gm5431	1.61E-14	5.498831079	3.82E-10
Ccdc190	1.61E-14	5.083793579	3.82E-10
4930515L19Rik	1.34E-13	5.176902984	3.17E-09
Gm47710	1.35E-13	4.913868578	3.19E-09
Mccc1os	6.29E-12	5.013404252	1.49E-07
Gm50069	4.78E-11	5.913868578	1.13E-06
Gm9903	4.81E-11	5.498831079	1.14E-06
Gm33914	4.81E-11	5.498831079	1.14E-06
C330019F10Rik	4.81E-11	5.498831079	1.14E-06
Gm20711	3.22E-10	4.498831079	7.64E-06
Rpa3	1.43E-09	4.176902984	3.40E-05
A630014C17Rik	5.98E-09	5.498831079	0.000141745
Tgfb3	6.02E-09	5.083793579	0.000142774
Gm16553	5.35E-08	3.913868578	0.001268227
Angpt4	1.52E-07	5.176902984	0.003596652
Ckmt2	1.53E-07	4.761865485	0.003625517
Dusp13	1.53E-07	4.761865485	0.003625517
Epcam	1.53E-07	4.761865485	0.003625517
Gm34081	1.54E-07	4.498831079	0.003640025
Tubb6	1.54E-07	4.498831079	0.003640025
Gm14643	7.19E-07	3.591940483	0.017043893
Styxl1	1.26E-06	3.020783782	0.029904424
Nlrp9b	1.54E-06	4.913868578	0.036610077
Arhgap33	1.55E-06	4.691476157	0.036735039
Mgst3	1.56E-06	4.498831079	0.036923244
Chrnb2	1.97E-06	4.176902984	0.046745746
**Per1^{cLKO} Fast Hepatocyte Fgf21^{High} vs Fgf21^{Low}**			
Clvs1	1.59E-11	5.38114108	3.78E-07
Nsg1	1.59E-11	5.38114108	3.78E-07
Shisa6	1.01E-07	4.38114108	0.00239928
Usp11	7.65E-07	3.018571001	0.018128874
Gm14302	1.85E-06	3.533144174	0.043753605
Npr3	1.85E-06	3.533144174	0.043753605

Table S3. Primer sequence used in this paper. Related to STAR Methods.

Target	Species	Forward (5'-3')	Reverse (5'-3')
Actin	Mouse	GATTACTGCTCTGGCTCCTAG	GACTCATCGTACTCCTGCTTG
Atf1	Mouse	GATTCCCACAAGAGTAACACGAC	CCTATGCTGTCAGATGAGTCCT
Bmal1	Mouse	TGACCCTCATGGAAGGTTAGAA	GGACATTGCATTGCATGTTGG
Clock	Mouse	AGAACTTGGCATTGAAGAGTCTC	GTCAGACCCAGAATCTTGGCT
Cry1	Mouse	CACTGGTTCCGAAAGGGACTC	CTGAAGCAAAAATCGCCACCT
Cyp4a14	Mouse	TTTAGCCCTACAAGGTACTTGGA	GCAGCCACTGCCTTCGTAA
Esrrg	Mouse	AAGATCGACACATTGATTCCAGC	CATGGTTGAACTGTAACTCCCAC
Fgf21	Mouse	CTGCTGGGGGTCTACCAAG	CTGCGCCTACCACTGTTCC
G6pc	Mouse	CGACTCGCTATCTCCAAGTGA	GTTGAACCAGTCTCCGACCA
Hmgcs2	Mouse	CCGTATGGGCTTCTGTTCAG	AGCTTTGTGCGTTCCATCAG
Pdk4	Mouse	AGGGAGGTCGAGCTGTTCTC	GGAGTGTTCACTAAGCGGTCA
Per1	Mouse	TCCCCTATTCGCTTCTGTGC	GCAGCAATCGATGGATCTGC
Per2	Mouse	GAAAGCTGTCACCACCATAGAA	AACTCGCACTTCCTTTTCAGG
Pgc1a	Mouse	ACACCGCAATTCTCCCTTGT	CGGCGCTCTTCAATTGCTTT
Ppara	Mouse	TGGTTCCTGGTGCCGATTTA	ACTAGCATCCCACTTAATTATGTATCT
Vnn1	Mouse	CTTTCCTCGCGGCTGTTTAC	CCTCCAGGTATGGGTAGATCGT
